# Self-supervised learning on millions of primary RNA sequences from 72 vertebrates improves sequence-based RNA splicing prediction

**DOI:** 10.1093/bib/bbae163

**Published:** 2024-04-11

**Authors:** Ken Chen, Yue Zhou, Maolin Ding, Yu Wang, Zhixiang Ren, Yuedong Yang

**Affiliations:** School of Computer Science and Engineering, Sun Yat-sen University, Guangzhou, China; Peng Cheng Laboratory, Shenzhen, China; School of Computer Science and Engineering, Sun Yat-sen University, Guangzhou, China; Peng Cheng Laboratory, Shenzhen, China; Peng Cheng Laboratory, Shenzhen, China; School of Computer Science and Engineering, Sun Yat-sen University, Guangzhou, China; Key Laboratory of Machine Intelligence and Advanced Computing (Sun Yat-sen University), Ministry of Education, China

**Keywords:** language model, RNA splicing, self-supervised learning

## Abstract

Language models pretrained by self-supervised learning (SSL) have been widely utilized to study protein sequences, while few models were developed for genomic sequences and were limited to single species. Due to the lack of genomes from different species, these models cannot effectively leverage evolutionary information. In this study, we have developed SpliceBERT, a language model pretrained on primary ribonucleic acids (RNA) sequences from 72 vertebrates by masked language modeling, and applied it to sequence-based modeling of RNA splicing. Pretraining SpliceBERT on diverse species enables effective identification of evolutionarily conserved elements. Meanwhile, the learned hidden states and attention weights can characterize the biological properties of splice sites. As a result, SpliceBERT was shown effective on several downstream tasks: zero-shot prediction of variant effects on splicing, prediction of branchpoints in humans, and cross-species prediction of splice sites. Our study highlighted the importance of pretraining genomic language models on a diverse range of species and suggested that SSL is a promising approach to enhance our understanding of the regulatory logic underlying genomic sequences.

## INTRODUCTION

Ribonucleic acids (RNA) splicing is a fundamental post-transcriptional process in eukaryotic gene expression, which removes introns from primary transcripts and ligates exons into mature RNA products. Though the mechanism underlying RNA splicing is complex, a variety of studies have found that some key determinants of splicing are encoded in DNA sequences [1–3]. Therefore, deciphering splicing codes from RNA sequences by computational models is a promising approach and will facilitate the interpretation of genetic variants that affect RNA splicing [4].

Early studies mainly aimed to identify short sequence motifs related to splicing, including exonic splicing enhancers [5], branchpoints (BPs) [6] and other splicing factors [7] with statistical models. Benefiting from accumulated high-throughput sequencing data, subsequent studies were able to employ machine learning and deep learning models to directly predict RNA splicing events from primary sequences. For example, hexamer additive linear (HAL) [8] is a model trained on alternative splicing events from millions of random sequences for predicting the change of exon skipping and 5′ alternative splicing induced by genetic variants. Splicing-based analysis of variants (SPANR) [3] is a Bayesian network model for predicting the percent spliced in (PSI or Ψ) of alternatively spliced exons. More recently, deep learning models like MMSplice [9], SpliceAI [10] and Pangolin [11] employed deep convolutional neural networks to predict alternative splicing events, splice sites or splice site usage. These methods achieved superior performance as compared to earlier studies and have been widely utilized to analyze aberrant RNA splicing events caused by genetic variants. Though significant progress has been made in this field, there is still room for further exploration. For instance, state-of-the-art splicing models were developed to predict splice sites or alternative splicing from primary sequences, while evolutionary information was not considered. Furthermore, BP, an important splicing regulator located at the 3’ end of introns and involved in the recognition of acceptor sites, was less studied. Unlike splice sites, which can be reliably detected from RNA-seq data [12, 13], BPs are much more difficult to be identified [14], making it challenging to develop computational models for BP prediction due to the lack of adequate high-confidence dataset.

To alleviate the problem of insufficient data, the self-supervised learning (SSL) method utilized by large pretrained language models (pLMs) [15–17] can be adopted. A common form of SSL is masked language modeling (MLM) and it has already been adopted to develop pLMs of protein [18, 19], non-coding RNA (ncRNA) [20] and prokaryote genome [21] sequences. These models were pretrained on a large number of sequences from a diverse range of species and thereby captured the evolutionary information that is critical for sequence-based modeling. However, these models cannot be directly applied to RNA splicing because eukaryotic protein-coding RNA sequences are very different from ncRNAs or prokaryote genome sequences. Though there are genome pLMs like DNABERT [22] and LOGO [23], they were pretrained on only the human genome, and thus it remains unclear whether pLMs pretrained on sequences from more species could improve sequence-based RNA splicing prediction.

Here, we developed a primary RNA language model, SpliceBERT, and used it to study RNA splicing. SpliceBERT was pretrained by MLM on over 2 million RNA sequences from 72 vertebrates. Compared to pLMs for only the human genome, SpliceBERT can effectively capture evolutionary conservation from primary sequences. The hidden states and attention weights generated by SpliceBERT can reflect the biological property of splice sites. Additionally, the context information from SpliceBERT is able to distinguish variants with different effects on RNA splicing. These findings suggest that SSL on diverse species is beneficial to learn biologically meaningful representations from sequences. As a result, SpliceBERT was shown effective to predict BPs in humans and splice sites across species. The SpliceBERT model is available at https://github.com/biomed-AI/SpliceBERT.

## METHODS

### Pretraining SpliceBERT on vertebrate primary RNA sequences

#### Pretraining dataset

We collected primary RNA sequences of 72 vertebrates for pretraining. The reference genomes and gene annotations were downloaded from UCSC genome browser [24] and the genome assembly versions are listed in Supplementary Table S3. RNA transcripts were extracted from the reference genomes using ‘bedtools getfasta’ [25] based on gene annotations and overlapping transcripts were merged to avoid redundancy. In this way, we constructed a dataset including over 2 million sequences and approximately covering 65 billion nucleotides. We reserved 50 000 randomly selected sequences for validation and pretrained SpliceBERT on the remaining sequences.

#### Tokenization

Existing genomic sequence language models mainly adopted the *k*-mer [22, 23, 26] or the Byte Pair Encoding (BPE) [27] tokenization. Here, we simply encoded each nucleotide (A, G, C, T/U) as a token for the ease of obtaining hidden states and attention weights of individual nucleotides. A ‘[CLS]’ (classification) token and a ‘[SEP]’ (separator) token were padded to the beginning and the end of each sequence, respectively, as a routine operation in BERT-style tokenizers [16].

#### Model architecture

SpliceBERT is based on the BERT [16] architecture, consisting of six Transformer [28] encoder layers. The hidden layer size and attention head number are 512 and 16, respectively. Positional information is encoded by absolution position embedding, and the maximum length of input sequence is set to 1024. SpliceBERT has about 19.4 million learnable parameters. The inputs to SpliceBERT are primary RNA sequences with the nucleotides (N/A/C/G/T) being converted to integer tokens, and the outputs are embeddings of nucleotides that can be used to make predictions in pretraining and downstream tasks.

#### MLM

We pretrained SpliceBERT by MLM in a self-supervised manner. Specifically, 15% of the nucleotides in each sequence input to SpliceBERT were randomly selected. Eighty percent and 10% of the selected nucleotides were replaced with the mask token (‘[MASK]’) and random nucleotides, respectively, and the rest of the selected nucleotides remained unchanged. During pretraining, SpliceBERT was trained to predict the correct type of the selected nucleotides, by which it will learn the dependencies between the nucleotides and capture the logic of RNA sequences.

#### Model implementation and pretraining

SpliceBERT was implemented in PyTorch (v1.9) with Huggingface transformer (v4.24.0) and Flash attention library [29]. We adopted cross-entropy (CE) loss as the objective:


$$ \mathrm{CE}\left(\boldsymbol{p},\boldsymbol{q}\right)=-{\sum}_i^C{p}_i\log \left({q}_i\right), $$

where


$$ {q}_i=\frac{e^{y_i}}{\sum_j^C{e}^{y_j}} $$

where *C* is the number of token types and $\boldsymbol{p}\in{\left\{0,1\right\}}^C$ and $\boldsymbol{y}\in{\mathcal{R}}^C$ are the true label and predicted token type distribution (in logit-scale) of each token, respectively. The AdamW [30] optimizer was used to update the weights in the model with an initial learning rate of 0.0001. The learning rate will be halved when validation loss stopped decreasing for three consecutive epochs. The model was trained in automatic mixed precision mode to reduce memory consumption and speed up model training. To improve training efficiency, we pretrained SpliceBERT in two stages. In the first stage, it was pretrained with sequences of a fixed length of 510 nt (512 nt with ‘[CLS]’ and ‘[SEP]’), which were randomly drawn from full-length RNA transcripts. In the second stage, we continued pretraining the model obtained from the first stage on sequences of variable length between 64 and 1024 nt (see Supplementary Methods for details). The first stage took 7 days on 8 NVIDIA V100 graphics processing units (GPUs), and the second stage took 3 days on 4 NVIDIA V100 GPUs. The CE loss on validation data finally converged to 1.02. Additionally, to assess the contribution of multi-species pretraining, we developed another model with identical architecture to SpliceBERT but was pretrained on only human RNA sequences, namely, SpliceBERT-human.

### Visualizing and clustering splice site embeddings

We investigated whether the nucleotide embeddings generated by SpliceBERT can characterize the nature of splice sites. To this end, positive samples (true splice sites) were collected from the canonical transcripts of each gene in GENCODE annotation (v41lift37) and the negative samples were defined as the decoy splice sites in the same transcripts, where the decoy splice sites refer to non-splice sites with a MaxEntScan [1] score above 3 [31]. Any samples that do not match canonical donor motif GT(U) or acceptor motif AG were discarded to eliminate the difference in nucleotide composition. Finally, we randomly sampled 5000 donor, acceptor, non-donor GT and non-acceptor AG sites, respectively, and generated the nucleotide embeddings by SpliceBERT. The embeddings of each GT/AG site were flattened into a 1024-dimension vector and then reduced to 128-d by principal component analysis (PCA) for Uniform Manifold Approximation and Projection (UMAP) [32] visualization. In addition, to mitigate the known drawback of UMAP for dimension reduction [33], we clustered the samples using the Leiden algorithm and quantitatively evaluate the clustering results by normalized mutual information (NMI) score. Here, the programming interfaces of PCA, UMAP and Leiden algorithms are implemented in the Scanpy package (v1.9) [34]. For comparison, we conducted the same analysis on the embeddings generated by SpliceBERT-human, DNABERT and one-hot encoding (N: [0, 0, 0, 0], A: [1, 0, 0, 0], C: [0, 1, 0, 0], G: [0, 0, 1, 0], T: [0, 0, 0, 1]) [35, 36], respectively. To be noted, we only show the optimal results of DNABERT and OHE because the value of *k* in DNABERT’s tokenization and sequence length in OHE significantly influence clustering and visualization, as shown in Supplementary Figures S1 and S2. Another RNA language model, RNA-FM [20], was not used for comparison because it was pre-trained only on mature non-coding RNAs, which are distinct from primary RNA and coding sequences, and thus cannot produce embeddings for characterizing splice sites (Supplementary Figure S3).

### Analyzing attention weights between splice sites

The self-attention module in each Transformer layer transforms the input feature map $\left(\boldsymbol{h}\in{R}^{L\times d}\right)$ of a sequence into a key and a query ($\boldsymbol{K},\boldsymbol{Q}\in{R}^{L\times{d}_k}$) with two learnable linear projections ${\boldsymbol{W}}_{\boldsymbol{q}},{\boldsymbol{W}}_{\boldsymbol{k}}\in{\mathrm{R}}^{\mathrm{d}\times{\mathrm{d}}_{\mathrm{k}}}$, respectively:


$$ \boldsymbol{K}=\boldsymbol{h}{\boldsymbol{W}}_k,\boldsymbol{Q}=\boldsymbol{h}{\boldsymbol{W}}_q $$

where *d* is the dimension of hidden size in Transformer layers, $L$ is the length of input sequence and ${d}_k$ equals to $d/h$(h is the number of attention heads, which is 16 in SpliceBERT). The dot product of *Q* and *K* is the attention matrix:


$$\boldsymbol{A}= \mathrm{softmax}\left(\frac{\boldsymbol{Q}{\boldsymbol{K}}^T}{\sqrt{d_k}}\right)\in{R}^{L\times L}.$$

The softmax function was applied by row, and thus, the attention weights in each row sum up to 1 [28]. For simplicity, the maximum attention weights across different attention heads were taken in our analysis. Each element ${a}_{ij}$ in A could be regarded as the association of the *j*-th token to the $i$-th token (note that ${a}_{ij}$ is not necessarily equal to ${a}_{ji}$). In our analysis, we averaged ${a}_{ij}$ and ${a}_{ji}$ to represent the attention between $i$ and $j$. When analyzing the attention weights of donor and acceptor sites, we took the average weights of the two nucleotides in intron and the one nucleotide in exon.

In our analysis, we focused on the introns less than 800 nt as SpliceBERT can only process sequences up to 1024 nt, which covers about 33% (78 252/235 039) of the introns in canonical transcripts based on GENCODE v41lift37 annotation.

### Assessing the impact of genetic variants using KL divergence

In MLM, SpliceBERT predicts a nucleotide type probability distribution for each token. The predicted probability distribution was then utilized to estimate the impact of variants on RNA splicing in a zero-shot manner. Under such a setting, we directly applied the pre-trained model on sequences with and without variants without any supervised fine-tuning on labeled datasets. Specifically, the predicted nucleotide type distribution of the $i$th nucleotide in a reference sequence can be represented as ${P}_i=\left({p}_A,{p}_C,{p}_G,{p}_T\right)$, where ${p}_A,{p}_C,{p}_G$ and ${p}_T$ sums up to 1. When a variant occurs in the sequence, it will introduce perturbations to the model’s output, resulting in the probability distribution of the nucleotide $i$ change from ${P}_i^{ref}$ to ${P}_i^{alt}$. Then, the change of distribution can be measured by Kullback–Leibler (KL) divergence:


$$ {D}_{KL}\left({P}^{alt}\Big\Vert{P}^{ref}\right)=\sum_{x\in \left\{A,C,G,T\right\}}{p}_x^{alt}\mathit{\log}\frac{p_x^{alt}}{p_x^{ref}}. $$

The probability values were clipped to be between $\left[{10}^{-6},1\right]$ to avoid division by zero error. We recruited two datasets of variants associated with RNA splicing (MFASS and Vex-seq) to illustrate the effectiveness of our approach. The MFASS dataset includes 27 733 single-nucleotide variants (SNVs) within or around exons. The SNVs that largely decrease exon splicing efficiency ($\Delta \Psi$ < −0.5, $\Psi :$PSI/percent spliced in, *n* = 1050) were considered as splice-disrupting variants (SDVs) [37]. The Vex-seq dataset includes 1971 SNVs with experimentally identified $\Delta \Psi$ and the SNVs with $\Delta \Psi <-0.24$ (top 5%, *n* = 98) were defined as SDVs. The evaluation was formulated as a binary classification problem (SDVs versus non-SDVs). The KL divergence was utilized to prioritize the impact of variants, and thus, metrics like precision–recall curves can be plotted based on the labels and predicted scores of variants.

### Finetuning SpliceBERT for predicting BP

SpliceBERT was finetuned on a human BP dataset [38] (the Mercer’s dataset) for predicting BP sites. The Mercer’s dataset includes BPs of high confidence (HC-BP) and low confidence (LC-BP) identified in the human transcripts. Following previous studies [38, 39], we focused on the 18–44 nt region upstream of splice acceptors (where BPs typically located) and recruited the HC-BP (*n* = 55 739) and non-BP (*n* = 921 518) sites within the focused regions as positive and negative samples, respectively. The input to SpliceBERT is a 510 nt sequence that covers both the up- and downstream of the 18–44 nt upstream of acceptors, and the hidden states in the last Transformer layer of SpliceBERT were fed to a single-layer fully connected neural network to predict BPs. To improve prediction efficiency, multiple sites in a single sequence can be predicted by SpliceBERT simultaneously. The nested cross-validation (CV) strategy was employed to finetune and evaluate SpliceBERT. The samples were split into 10 folds by chromosomes, and therefore, the samples from the same chromosome were always kept in the same CV fold. In each training epoch, each of the 10 CV folds was reserved, in turn, as the test dataset, and the other folds were used to finetune and validate SpliceBERT (eight folds for training and one fold for validating). The optimal number of training epochs was determined according to the average performance on validation data across the 10 folds, and the training process will be terminated when the CV performance stopped to improve for 10 epochs. The final performance is measured by the average precision (AP) score on the 10 test CV folds. We compared SpliceBERT to Branchpointer [38, 40], LaBranchoR, DNABERT, SpliceBERT-human and RNA-FM. The predictions of Branchpointer were generated using its scripts, and the results of other models were obtained by training and testing them using the same nested CV scheme to SpliceBERT. The results of RNA-RM are only shown in Supplementary Table S5 because RNA-FM was designed for mature non-coding RNA sequences and thus, it is not appropriate to apply RNA-FM to coding transcripts directly.

### Finetuning SpliceBERT for predicting splice sites

SpliceBERT was finetuned on the Spliceator dataset [41] to predict splice sites across species. The Spliceator dataset curated error-free splice sites from over 100 eukaryotic species and provided five independent test datasets from *Homo sapiens* (human), *Danio rerio* (zebrafish), *Drosophila melanogaster* (fruit fly), *Caenorhabditis elegans* (worm) and *Arabidopsis thaliana* (Arabidopsis). Because human splice sites are also included in training data, only the four non-human test datasets were used to evaluate the model’s performance for cross-species splice site prediction. Each sample in the dataset is a 600 nt/400 nt sequence centered on a splice/non-splice site. To ensure the consistency of samples, sequences of 600 nt were truncated to 400 nt. We finetuned SpliceBERT as a sequence classification task, feeding the hidden state of the ‘[CLS]’ token in the last layer to a two-layer fully connected neural network to make predictions. To make our results comparable to those reported in the Spliceator paper, we followed the 10-fold cross-validation scheme described in the study to finetune SpliceBERT and test it on the same independent datasets. Baseline models for comparison include SpliceBERT-human, DNABERT, SpliceAI-400 nt, Spliceator, DSSP, MaxEntScan and SpliceFinder. The results (measure by F1 score) of Spliceator, DSSP, MaxEntScan, SpliceFinder and NNSplice were directly taken from the Spliceator paper, and the results of the other models were obtained by training and testing on the same datasets. To be noted, the state-of-the-art SpliceAI [10] model (SpliceAI-10 k) was not used for comparison because it takes ultra-long sequences of at least 10 001 nt, which largely exceeds the maximum length that other models can process ($\le$1024 nt). Thus, we only compared SpliceAI-400 nt with other models to fairly assess their performance to make prediction from short sequences.

## RESULTS

### MLM captures evolutionary conservation information

**Figure 1:**
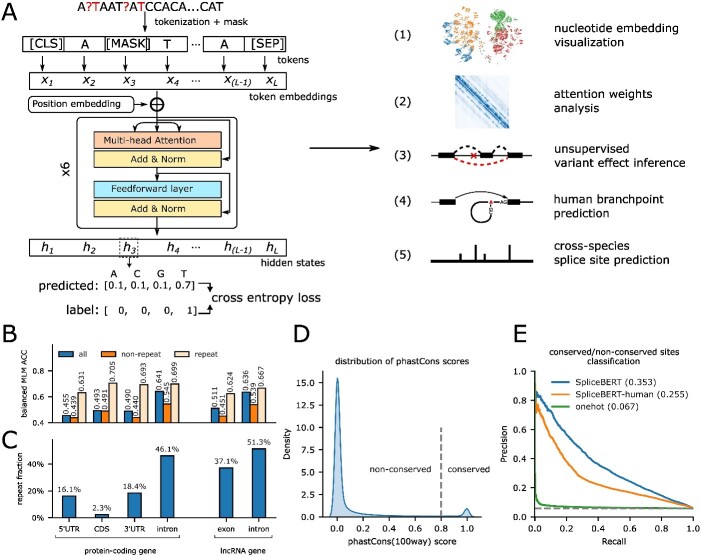
Pretraining SpliceBERT on primary RNA sequences by masked language modeling. (**A**) The structure of SpliceBERT and its applications in this study. (**B**) Balanced accuracy of masked token prediction (MLM ACC) in repetitive/non-repetitive regions of different functional genomic regions. (**C**) The fraction of repeats in different functional regions of protein-coding and lncRNA transcripts. (**D**) The distribution of phastCons (100way) score in transcripts. The cut-off of conserved/non-conserved sites is set to 0.8. (**E**) The precision–recall curves of logistic regression models for distinguishing between nucleotides at conserved and non-conserved sites based on nucleotide embeddings from SpliceBERT, SpliceBERT-human and one-hot encoding (UTRs: untranslated regions, CDSs: coding sequences, lncRNA: long non-coding RNA).

SpliceBERT is a pretrained primary RNA language model (LM) (Figure 1A), which was developed based on the Bidirectional Encoder Representations from Transformers (BERT) [16, 28] architecture and pretrained by MLM on over 2 million RNA sequences from 72 vertebrates. The inputs to SpliceBERT are primary RNA sequences, including both exonic and intronic regions of genes. Each nucleotide is regarded as an individual token for the model and the maximum length of input sequence is 1024 nt. For MLM pretraining, SpliceBERT was set to predict the type of nucleotides that were masked in the input sequences, by which it could learn dependencies between nucleotides in a self-supervised manner. Figure 1B illustrates that the balanced accuracy (ACC) for nucleotide type prediction in MLM is 0.641 (0.636) and 0.493 (0.511) for introns and exons, respectively, in coding (non-coding) genes. The higher ACC in introns is likely attributed to its higher sequence repeat contents: 46.1%/51.3% in coding/non-coding genes versus 2.4%/37.1% in coding/non-coding genes (Figure 1C). Similar results can be found in different functional regions when we analyzing repetitive/non-repetitive regions separately or conducted the same analysis with DNABERT (Supplementary Figure S4). These observations indicated that the pattern of non-coding sequences is easier to be captured due to enrichment of repeated sequences, which are composed of simple short tandem repeats or low-complexity regions [42].

It is of interest to investigate whether MLM pretraining could enable SpliceBERT to capture evolutionary information because most methods for identifying evolutionarily conserved elements are based on multiple sequence alignment (MSA) [43], which is time-consuming. To this end, we extracted hidden states in the last encoder layer of SpliceBERT and fed them to a logistic regression (LR) model to see if the embeddings obtained by SSL can be directly exploited to distinguish between conserved (phastCons $\ge$ 0.8) and non-conserved (phastCons $<$ 0.8) sites (Figure 1D) (see Supplementary Methods). Though SpliceBERT leveraged only 72 vertebrates in pretraining while phastCons was derived from 100 vertebrates, the LR model based on SpliceBERT embeddings achieved an AP score of 0.353, outperforming the baseline models that rely on SpliceBERT-human (AP = 0.255), one-hot encoding (AP = 0.067) and random prediction (AP = 0.058). The results demonstrated that MLM pretraining can capture evolutionary information, and this ability can be enhanced by augmenting the pretraining data with sequences from diverse species. The ability is potentially beneficial for sequence-based modeling tasks, and we thus conducted further analysis in the following sections to illustrate this point (summarized in Supplementary Table S6).

### Nucleotide embeddings learned by SpliceBERT characterize the property of splice sites

**Figure 2:**
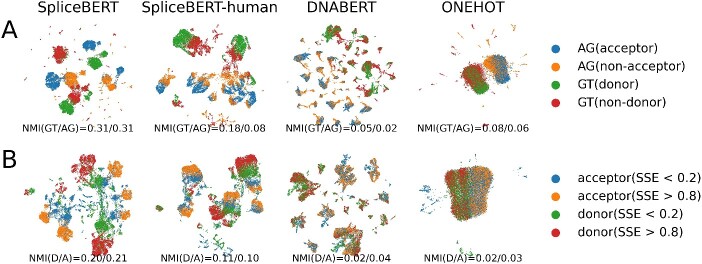
Investigating the nucleotide embeddings of splice sites. UMAP visualization of nucleotide embeddings generated by different methods for (**A**) canonical splice sites (GT/AG) and non-splice GT/AG sites and (**B**) splice sites of high and low usage in K562 estimated by SpliSER. For SpliceBERT and SpliceBERT-human, the hidden states of the last transformer encoder layer were used as nucleotide embeddings (NMI: normalized mutual information, D: donor, A: acceptor). The sample size of each group is 5000.

The nucleotide embeddings learned by SpliceBERT were studied to assess whether they were able to characterize the biological properties of splice sites. To achieve this, canonical splice sites (SS) and non-splice GT/AG sites (NSS) were collected from the human genome and the nucleotide embeddings of them were generated using SpliceBERT and three baseline methods (SpliceBERT-human, DNABERT and one-hot). These embeddings were visualized by the UMAP [32] algorithm and clustered by the Leiden [44] algorithm. As shown in Figure 2A, SpliceBERT achieved the highest normalized mutual information (NMI) score (NMI = 0.31/0.31 for GT/AG, respectively) for distinguishing between SS and NS, surpassing SpliceBERT-human (NMI = 0.18/0.08), DNABERT (NMI = 0.05/0.02) and one-hot encoding (NMI = 0.08/0.06). Though DNABERT and SpliceBERT-human were both pretrained on the human data, the lower NMI of DNABERT may stem from its *k*-mer tokenization strategy, which tends to generate more clusters as the value of *k* increases (Supplementary Figure S1). Next, we conducted the same analysis on splice sites of high (splice strength estimation/SSE > 0.8) and low (SSE < 0.2) strength estimated by SpliSER [45] in the K562 cell line (see Supplementary Materials). This presents a more challenging scenario as all the samples are authentic splice sites. As expected, the NMI scores decreased for all the models (Figure 2B), while SpliceBERT still achieved the highest NMI score (NMI = 0.20/0.21 for donor/acceptor). Besides, the embeddings of splice sites in the same class can further be clustered into subgroups, which is likely due to the distinct motif patterns (Supplementary Figure S5). These findings indicated that SpliceBERT trained on a diverse of species is more powerful to capture the sequence determinants of splice sites than human-only pLMs.

In addition to the last layer’s hidden states (as shown in Figure 2), the hidden states in layers 2–5 in SpliceBERT are also informative for distinguishing SS from NSS (Supplementary Figure S6). The optimal performance was achieved by hidden states in the 4th layer but not the last layer (the 6th layer). This is probably because the hidden states of the last layer are mostly related to predicting the masked tokens during pretraining [46], while the intermediate layers preserve more contextual information.

### Attention weights in SpliceBERT correlate with donor–acceptor dependencies

**Figure 3:**
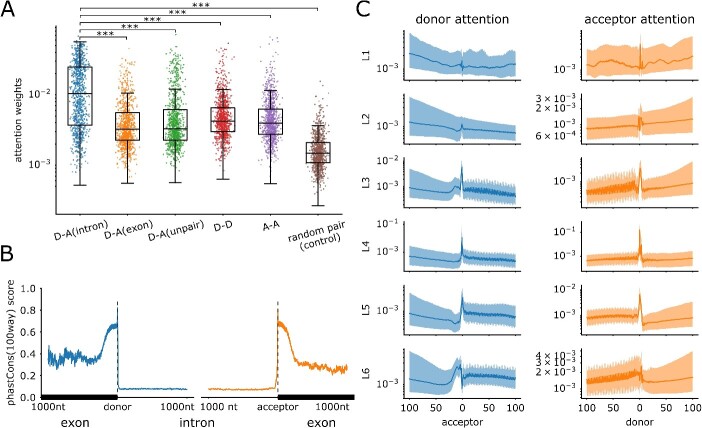
Analyzing the attention weights in SpliceBERT. (**A**) The distribution of attention weights between donors and acceptors within the same intron [D-A(intron)], within the same exon [D-A(exon)], across different intron/exon [D-A(unpair)], among donors (D-D) and among acceptors (A-A). A control group of randomly paired nucleotides is also shown (random pair). The average attention weights across all the six layers in SpliceBERT were used. The statistical significance was assessed by one-sided Mann–Whitney U test. (**B**) The distribution of phastCons (100way) scores around donors and acceptors within 1000 nt (from the same introns). (**C**) The distribution of donors’ and acceptor’s attention weights around acceptors and donors, respectively, in each Transformer layer. The 25, 50 (median) and 75 percentile of attention weights across different samples are shown (D: donor, A: acceptor, L: layer, ^***^: *P*-value < $1\times{10}^{-16}$).

SpliceBERT is mainly consisted of a stack of Transformer encoders, which utilize self-attention module to capture long-range dependencies in the sequence. Intuitively, the self-attention module is expected to capture the association between different tokens (nucleotides) in the input RNA sequences. Therefore, it is of interest to investigate whether the attention weights in SpliceBERT could reflect the functional relationship between donor and acceptor splice sites. Concretely, we compared the attention weights between various different donor/acceptor site combinations, such as donor-acceptor pairs from the same or different introns/exons (Supplementary Figure S7) or random site pairs (control group). To account for the potential influence of distance on attention weights, we sampled approximately 1000 pairs in each group and ensured that distance distribution was comparable across different groups. As shown in Figure 3A, the attention weights between donor/acceptor pairs were consistently higher than those between random pairs, which was likely due to the higher evolutionary conservation around splice sites than the other sites (Figure 3B). More importantly, donor–acceptor pairs from the same introns also achieved higher attention weights than other donor/acceptor combinations (Figure 3A, *P*-value < ${10}^{-6}$ by Mann–Whitney U test, Cohen’s d effect size [47] ranges between 0.83 and 1.04, Supplementary Table S1), and the attention weights of alternative splice sites exhibited the same trend to PSI estimated from RNA-seq samples (Supplementary Methods and Figure S8). This implied that SpliceBERT captured the functional association between donors and acceptors from the same introns, which is in line with the intron-centric nature of RNA splicing [48, 49] and the enrichment of conserved complementary regions in the ends of introns [50]. To investigate the contribution of different encoder layers, we analyzed the attention weights between donor-acceptor pairs from the same introns from layer 1 to layer 6, respectively. The attention weights of the donor (acceptor) sites were aligned with respect to the acceptor (donor) sites and the median values across all the samples were calculated. As illustrated in Figure 3C, attention weight peaks can be observed around the acceptor/donor sites in the 3rd, 4th and 5th layer, which is 1.9, 16.1 and 1.7 times that of background weights (averaged across the entire regions, Supplementary Table S1). In contrast, the weights of donors at acceptors are only 0.7, 0.5 and 0.7 times the average attention in the 1^st^, 2^nd^ and 6^th^ layer, respectively. This indicates that the attention weights in layers 3–5 can better capture donor-acceptor associations, which is consistent with the observation in hidden states (Supplementary Figure S6). Taken together, the attention weights in SpliceBERT can reflect the functional associations between splice donors and acceptors.

### SpliceBERT-derived context information improves zero-shot variant effects prediction

**Figure 4:**
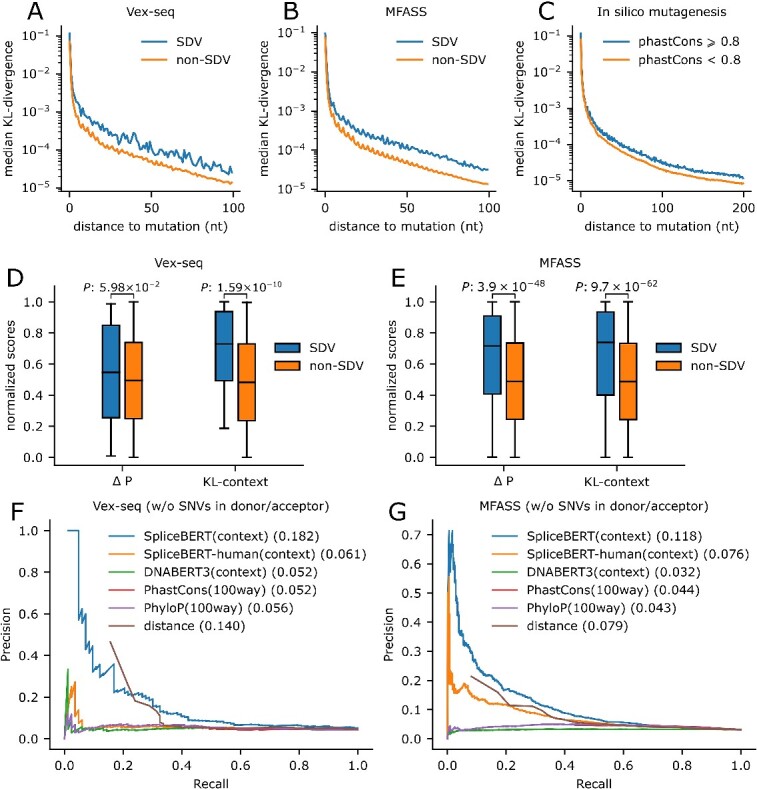
Applying SpliceBERT to zero-shot variant effect prediction for RNA splicing. The median value of KL-divergence between the MLM logits of wild-type and mutant sequences of splice-disrupting (SDV, *n* = 98/1050 in Vex-seq/MFASS) and non-splice-disrupting (non-SDV, *n* = 1873/26 683 in Vex-seq/MFASS) variants in (**A**) Vex-seq and (**B**) MFASS. (**C**) The median value of KL divergence around variants at conserved and non-conserved sites. The boxplot of normalized (scaled to 0–1 range) delta-logit ($\Delta$P) and KL-context score of SDV and non-SDV group in (**D**) Vex-seq and (**E**) MFASS. The precision–recall curves of SpliceBERT and baseline methods for classifying SDV and non-SDV samples in (**F**) Vex-seq and (**G**) MFASS.

We next utilized SpliceBERT to interpret the effects of variants on RNA splicing in a zero-shot manner. This was achieved by measuring the Kullback–Leibler (KL)-divergence between the predicted nucleotide type distributions of nucleotides around variants in reference and alternate sequences. Our analysis of two splicing-related variant datasets (MFASS [37] and Vex-seq [51]) suggested that splice-disrupting variants (SDVs) generally have a more significant impact on the predicted nucleotide type distributions of adjacent nucleotides compared to non-SDVs (Figure 4A and B). Furthermore, we examined the variants at conserved (phastCons $\ge$ 0.8) and non-conserved (phastCons $<$ 0.8) sites, as evolutionarily conserved regions in genomic sequences typically have critical functions. As expected, variants at conserved sites tend to induce greater change in predicted nucleotide type distributions than those at non-conserved sites (Figure 4C). These findings indicated that the predicted distributions of flanking nucleotide around variants can serve as indicators of the functional effects of genetic variants.

Therefore, we took the sum of KL-divergence (in logarithm scale) within 100 nt up- and downstream of each variant (KL-context score) for inferring the variants’ effects on RNA splicing. Compared with the metric adopted by previous studies ($\Delta P$) [52, 53], which measures the change of allele logit, the difference in median KL-context score between SDVs and non-SDVs is larger in Vex-seq and MFASS (Figure 4D and E), respectively. Next, we quantified the performance of KL-context scores derived from different pLMs (SpliceBERT, SpliceBERT-human and DNABERT), phastCons, phyloP and distance to splice sites on Vex-seq and MFASS. Here, the SNVs at donor/acceptor sites were excluded due to their high prevalence as SDVs (Vex-seq: 68.2%, MFASS: 80.7%, Supplementary Figure S9). As shown in Figure 4F and G, SpliceBERT-derived KL-context scores consistently outperformed human-only pLMs (SpliceBERT-human and DNABERT), conservation scores (phyloP and phastCons) and the distance from variants to splice sites. These results demonstrated that incorporating the context information derived from SpliceBERT can improve zero-shot inference of variant effects on RNA splicing.

### SpliceBERT improves BP prediction

**Figure 5:**
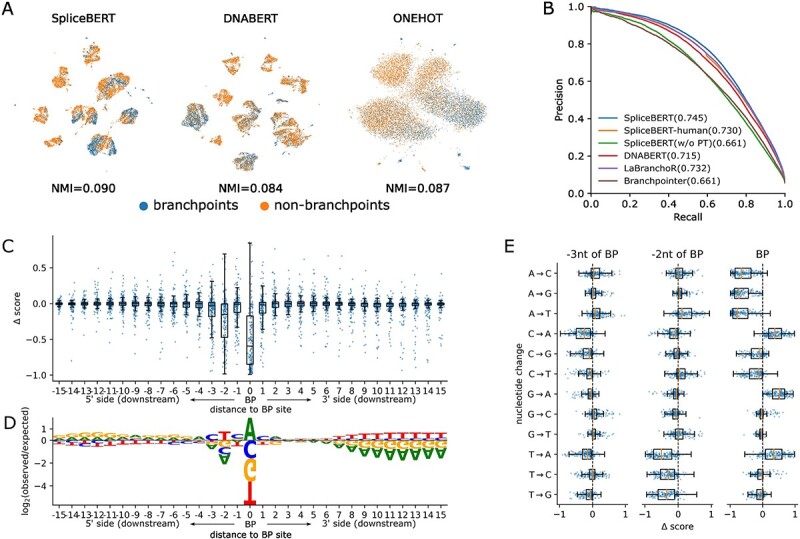
Predicting human branchpoint sites using SpliceBERT. (**A**) The UMAP visualization of BP and non-BP site embeddings. The NMI scores were calculated and averaged for each sequence motif, respectively. (**B**) The precision–recall curves of the models for predicting BPs. (**C**) The impact of variants at different sites on SpliceBERT predicted BP scores and (**D**) the sequence consensus (see Supplementary Materials) around branchpoints. (**E**) The predicted change of BP score induced by different mutation types at the BP sites and 2 and 3 nt upstream of BP sites.

In addition to the splice site, BP is another essential splicing regulator, which involves splice acceptor identification [54]. We first generated the embeddings of BP and non-BP sites that conform to the typical YTNAY motif [14] (Supplementary Figure S10) and visualized them by UMAP. The embeddings generated by SpliceBERT achieved an NMI score of 0.090 to distinguish BPs from non-BPs via Leiden clustering (Figure 5A), which is much lower than the results for splice sites (Figure 2). The performance of DNABERT and one-hot encoding is even worse (NMI = 0.084/0.087). This indicates that BPs are difficult to be characterized solely through MLM pretraining, possibly because BP sequences are highly degenerate [14] and are usually difficult to be accurately predicted from sequences [54].

To enhance sequence-based BP prediction, SpliceBERT was finetuned on Mercer’s dataset [54] that includes BPs identified in the human genome. We adopted the 10-fold nested cross-validation strategy to avoid over-fitting and make full use of the samples for evaluation. SpliceBERT achieved an AP score of 0.745, outperforming SpliceBERT-human, DNABERT, LaBranchoR [40] and Branchpointer [38] by 2.1%, 4.2%, 1.8% and 12.7%, respectively (Figure 5B). Additionally, the pretraining process is found to be indispensable, as the AP score of the SpliceBERT model trained from scratch without pretraining (SpliceBERT w/o PT) is only 0.661. These results demonstrated that SpliceBERT can improve sequence-based prediction of human BPs. *In silico* mutagenesis (see Supplementary Materials) indicated that the variants that have a significant impact on BP prediction enriched within the range of 3 nt upstream to 1 nt downstream of the BP site ([BP-3, BP + 1]), consistent with the known BP motif (Figure 5C and D). The loss of adenine at BP sites significantly reduced predicted BP scores, and variant T > A and T > G at BP-2 can also largely decrease predicted BP scores (Figure 5E). These observations are consistent with the known patterns of pathogenic variants around BP sites as reported in Zhang *et al*.’s study [55].

### SpliceBERT improves cross-species splice site prediction

**Figure 6:**
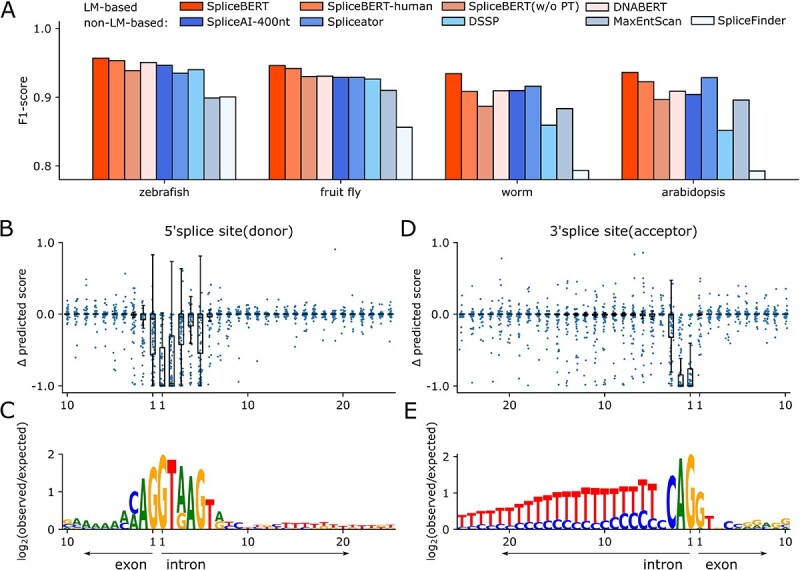
Predicting splice sites in different species using SpliceBERT. (**A**) The F1-score of SpliceBERT and baseline models on four species. The visualization of the impact of *in silico* mutagenesis variants on splice site scores for (**B**) donors and (**D**) acceptors and sequence consensus around splice sites (**C** and **E**). The *X*-axis of (**B**) and (**D**) indicates the distance from each nucleotide to the BP sites.

SpliceBERT was expected to achieve better performance in cross-species prediction since it was pretrained on more than 70 vertebrates. To address this, we finetuned SpliceBERT to predict splice site on the Spliceator [41] training dataset, which included splice sites in over 100 species, and tested it on test datasets from zebrafish, fruit fly, worm and Arabidopsis. The performance was measured by F1 score (Figure 6A, see Supplementary Table S2 for precision and recall). SpliceBERT achieved superior performance to baseline models on the test datasets (*P*-value <$0.05$, by two-sided paired *t*-test, Supplementary Table S2D). In particular, SpliceBERT surpassed that of SpliceBERT-human and DNABERT by an average of 1.6% and 2.1%, respectively. This suggests that SpliceBERT compares favorably to the models pretrained on only human sequences in cross-species prediction.

To confirm the prediction, *in silico* mutagenesis analysis was performed to compare the putative variant effects with the known pattern of SS. For splice donors, we observed that the variants leading to large change in predicted scores enriched in a 6 nt window around donors (1 nt in exon and 5 nt in intron, Figure 6B). Similarly, for splice acceptors, the variants in the last 3 nucleotides in the 3’ end of introns were found to have a substantial impact on predicted scores (Figure 6D). These observations were consistent with the known motif of splice sites [11], further validating our predictions.

## DISCUSSION

SSL, which has the ability to learn on unlabeled sequences [16, 18], presents a great opportunity to advance our understanding of genomic sequences. To assess how SSL can capture genetic information from genomic sequences, we collected primary RNA sequences from 72 vertebrates and developed a pre-trained language model, SpliceBERT, by masked language modeling on them. Benefiting from the pretraining data that cover a diverse range of species, SpliceBERT generated nucleotide representations that include evolutionary information and are able to characterize the biological properties of splice sites. As a result, SpliceBERT is shown able to improve sequence-based prediction of splice sites and BP sites. Although our model did not surpass baseline models by a very large margin, the superior performance of SpliceBERT to language models that pretrained on only the human genome can still demonstrate that multi-species pretraining is an effective approach for improving genomic language models.

Though previous studies have applied pLMs to genomic sequences [22, 23], they are limited to the human genome and focused on pLM’s performance on downstream tasks after finetuning, lacking a comprehensive analysis of the nucleotide representations learned by SSL. In this study, we performed an in-depth analysis of representations generated by SpliceBERT without finetuning and found that SSL itself is a powerful approach to capturing evolutionary and genetic information from large-scale genomic sequences. Besides, expanding the pretraining data with sequences from multiple species can further enhance SpliceBERT’s performance in both unsupervised and supervised evaluations. These findings indicate that pLMs have a great potential for computational genomics studies, as genomic sequence data are more abundant and easier to be obtained than other functional genomic data like transcription factor binding sites and histone modifications [56].

Although promising, the current study is meeting several limits. First, masked language modeling is still of limited accuracy (balanced ACC ranges between 0.45 and 0.65, Figure 1B) compared to a random model which is expected to achieve a balanced accuracy of about 0.25. This is likely due to the limited size of our model and pretraining dataset. With hundreds of primate and mammal genomes released in recent studies [57, 58], we will be able to enhance our model by expanding its size and the amount of pretraining data because the performance of pLMs usually scales up with the size of model and dataset [59]. The taxonomical tree can also be utilized to weight the species in training data to improve the model’s performance on a certain group of species that we are mostly interested in (e.g. human or mouse). Second, SpliceBERT was only pretrained on vertebrate RNA sequences, which make up only a small fraction of eukaryotes. More powerful genomic pLMs may focus on additional organisms, especially metagenomes [60]. How to improve the quality of pretraining data will be a critical issue as MLM on non-repetitive sequences is much more challenging than MLM on repetitive regions. Therefore, reducing the sampling frequency of repetitive regions may improve the training efficiency of genomic pLMs. Third, SpliceBERT can only process sequences no longer than 1024 nt, while prior studies have revealed that leveraging large-scale genomic information can boost the performance of many models in genomics [61–64], especially for splice sites [10]. The main difficulty in scaling SpliceBERT to longer sequences is the quadratic space complexity of the self-attention model with respect to the sequence length. It remains necessary to explore the use of self-attention modules with sub-quadratic complexity [65, 66] or convolutional networks [67] for developing more powerful and lightweight genomic language models. Finally, the pretraining is tissue/cell type–agnostic, whereas many biological processes often occur in a tissue/cell-specific manner [68]. This problem might be solved by the effective fusion of sequence embedding and experimental data.

**Key Points**

- SpliceBERT captures evolutionary conservation information through self-supervised learning on primary RNA sequences from 72 vertebrates.
- SpliceBERT generate nucleotide embeddings and attention weights correlate with the biological property of splice sites in an unsupervised manner.
- By self-supervised learning, SpliceBERT improves splice site and branchpoint prediction from short sequences.

## Supplementary Material

splicebert_supp_bbae163

SpliceBERT_Tables_BIB_v1_bbae163

## Data Availability

The source code of SpliceBERT is available at https://github.com/biomed-AI/SpliceBERT. The data and model weights for running the analysis in this manuscript are available at https://doi.org/10.5281/zenodo.7995778. The reference genomes and gene annotations of the 72 vertebrates were downloaded from UCSC Genome Browser https://hgdownload.soe.ucsc.edu/downloads.html, with the names listed in Supplementary Table S3. The annotation of repeats in human genome (hg19) was obtained through the UCSC Table Browser https://genome.ucsc.edu/cgi-bin/hgTables. The GENCODE genome and annotation of human were downloaded from https://www.gencodegenes.org/human/release_41.html and https://www.gencodegenes.org/human/release_41lift37.html. PhastCons and phyloP conservation scores are available at http://hgdownload.cse.ucsc.edu/goldenpath/hg19/phastCons100way/ and http://hgdownload.cse.ucsc.edu/goldenpath/hg19/phyloP100way/. The alignment files of RNA-seq in K562 were downloaded from https://www.encodeproject.org/, with accession IDs listed in Supplementary Table S4.

## Author Biographies

**Ken Chen** is a PhD student at School of Computer Science and Engineering, Sun Yat-Sen University. His research interests focus on developing computational models for genomics and computational biology.

**Yue Zhou** is an algorithm engineer at Peng Cheng Laboratory. Her research interests include deep learning, distributed machine learning, high-performance computing and artificial intelligence (AI) for science.

**Maolin Ding** is a graduate student at School of Computer Science and Engineering, Sun Yat-Sen University. His research interests include deep learning and bioinformatics.

**Yu Wang** is a professor at Peng Cheng Laboratory. His research interests include AI for systems biology, particularly foundation models in biomedicine research.

**Zhixiang Ren** is an associate research scientist at Peng Cheng Laboratory. His research interests include AI for science, high-performance artificial intelligence and scientific computing.

**Yuedong Yang** is a professor at School of Computer Science and Engineering, Sun Yat-Sen University. Currently, he focuses on integrating HPC and AI techniques for multi-scale biomedical research.
