# Supplementary material for: Self-supervised learning on millions of primary RNA sequences from 72 vertebrates improves sequence-based RNA splicing prediction
Source: Brief Bioinform. 2024 Apr 11;25(3):bbae163. doi: 10.1093/bib/bbae163 (PMC11009468; doi:10.1093/bib/bbae163)
Supplement: splicebert_supp_bbae163 [file splicebert_supp_bbae163.pdf]

Self-supervised learning on millions of primary RNA sequences from 72 vertebrates improves sequence-based RNA splicing prediction

Supplementary Materials

Ken Chen[1], Yue Zhou[2], Maolin Ding[1], Yu Wang[2], Zhixiang Ren[2,*], and Yuedong Yang[1,3,*]

[1]School of Computer Science and Engineering, Sun Yat-sen University, Guangzhou, Guangdong, China

[2]Pengcheng Laboratory, Shenzhen, Guangdong, China

[3]Key Laboratory of Machine Intelligence and Advanced Computing (Sun Yat-sen University), Ministry of Education, China

[*]To whom correspondence should be addressed. Email: yangyd25@mail.sysu.edu.cn, renzhx@pcl.ac.cn

Contents

1 Pre-training SpliceBERT **2**

2 Functional genic region, repetitive sequence and evolutionary conservation annotation **3**

3 Distinguishing conserved/non-conserved sites with nucleotide embedding **3**

4 Estimating splice site strength from RNA-seq samples **4**

5 Analysis of attention weights at alternative spliced sites **4**

6 In silico mutagenesis analysis **4**

7 Visualization of consensus sequences **4**

List of Figures

S1 UMAP of DNABERT embeddings 6
S2 UMAP of one-hot encoded embeddings 7
S3 UMAP of splice site embeddings generated by RNA-FM 8
S4 MLM ACC of DNABERT 8
S5 Analyzing Splice site clusters 9
S6 UMAP of nucleotide embedding from differnet layers 10
S7 Donor/acceptor combination 10
S8 Analyzing PSI from RNA-seq samples 11
S9 Zero-shot prediction of RNA splicing (all SNPs) 11
S10 Visualization of the consensus sequences around branchpoints 12
S11 PR curves of SpliceBERT in MLM 13

1 Pre-training SpliceBERT

We downloaded the latest reference genomes and gene annotations of vertebrates from the USCS Genome Browser[1] (Haeussler et al., 2019) in Jul. 2022, where the versions are available in Table S1. The genome files in uncompressed fasta format exceeds 150 GB and cannot be loaded into memory entirely. Therefore, we converted each chromosome to a numpy array (data type: `numpy.int8`) with one-hot encoding (N: 0, A: 1, C: 2, G: 3, T: 4) and saved them in `hdf5` format. Then, the transcript sequences can be randomly accessed from disk with genomic coordinates during model training. For pre-training, we sampled transcripts in proportion to their sequence length. Since the length of transcripts varies greatly and more than 80 % are longer than 1024nt, sequences fragments no longer than 1024nt were randomly drawn from full-length transcripts. When the selected fragments exceeded the boundary of the transcript, the flanking genomic sequences of the transcript will be padded. The sampling process was performed on the fly during pre-training, which means that samples used for pre-training were different in each epoch.

SpliceBERT was pre-trained in two stages. SpliceBERT was pre-trained on fixed-length sequences of 510nt in the first stage and was extended to sequences between 64nt and 1024nt in the second stage. Since SpliceBERT uses learnable position embeddings to encode positional information, it can not be directly applied to sequences longer than 510nt. To make the model converge faster, we adopted the hierarchical decomposition strategy proposed by (Su, 2020) to extend the absolute positional embeddings to a maximum of 1024nt. Briefly, we use $\boldsymbol{p}_1, \boldsymbol{p}_2, \cdots, \boldsymbol{p}_m, \boldsymbol{p}_{m+1}, \cdots, \boldsymbol{p}_n$ to represent the position embeddings in the model (e.g., $m = 510, n = 1024$ in our study), where $\boldsymbol{p}_1, \cdots, \boldsymbol{p}_m$ have been trained in the first stage. The hierarchical decomposition strategy decomposes the embeddings into:

$$\boldsymbol{p}_{(i-1)\times m+j} = \alpha \boldsymbol{u}_i + (1-\alpha)\boldsymbol{u}_j, \qquad i, j \in \{1, 2, \cdots, m\} \tag{1}$$

, where $\boldsymbol{u}_i = \dfrac{\boldsymbol{p}_i - \alpha \boldsymbol{p}_1}{1-\alpha}, \alpha \neq 0.5$ ($\alpha = 0.4$ by default). This strategy can speed up the pre-training in the second stage.

[1] `https://hgdownload.cse.ucsc.edu/goldenpath/`

2 Functional genic region, repetitive sequence and evolutionary conservation annotation

For functional genic region annotation, we annotated protein-coding and long non-coding RNA (lncRNA) transcripts against the GENCODE gene annotation (v41lift37, GRCh37/hg19, released in January 2022, mapped from GRCh38). In protein-coding genes, the genic regions include 5 prime untranslated regions (5'UTR), coding sequences (CDS), 3 prime untranslated regions (3'UTR) and introns. In lncRNA genes, the genic regions include exons and introns. Due to the existence of gene isoforms, the same locus may be annotated into multiple categories. The pre-processed region annotation was stored in a block compressed file (bgzip format) and thus can be randomly accessed by tabix(Li, 2011) wrapped in the selene(Chen, Cofer, Zhou, & Troyanskaya, 2019) package. For repetitive sequence (repeat) annotation, we downloaded the RepeatMasker annotation (hg19) in bed format from the UCSC Table browser, and employed "`bedtools intersect`" to count the proportion of repeats in different functional genic regions. For evolutionary conservation annotation, we downloaded the phastCons(Siepel et al., 2005) and phyloP(Pollard, Hubisz, Rosenbloom, & Siepel, 2010) conservation scores derived from multiple sequence alignments (MSA) of 99 vertebrate genomes to human genome (hg19) from UCSC. The conservation scores can be extracted from bigwig files using the pyBigWig(Ramírez et al., 2016) package.

3 Distinguishing conserved/non-conserved sites with nucleotide embedding

Nucleotide embeddings are represented by the hidden states in the last Transformer encoder layer by default. We trained logistic regression (LR) models that took nucleotide embeddings as input to distinguish between nucleotides at conserved (phastCons $\geqslant 0.8$) and non-conserved (phastCons < 0.8) sites. Specifically, the embeddings of nucleotides from 1000 randomly selected 510nt sequences were generated by SpliceBERT, and randomly split into a training dataset (80 %) and a test dataset (20 %). The nucleotides with unknown ("NaN") phastCons scores were excluded. Then, an LR model was fitted on the training data and then evaluated on the test dataset. No validation dataset was needed here because we simply adopted the default configuration to train LR models. SpliceBERT-human and one-hot encoding were used as baseline methods for comparison. SpliceBERT-human is the model with the same structure as SpliceBERT but was pretrained on only human RNA sequences. One-hot encoding is to encode each nucleotide plus its 250nt up- and downstream (501nt in total) with one-hot encodings (A: [1, 0, 0, 0], C: [0, 1, 0, 0], G: [0, 0, 1, 0], T: [0, 0, 0, 1], N: [0, 0, 0, 0]) and flatten into a 2004-dimension vector. We plotted precision-recall curves for the prediction of these models and quantified their performance by the average precision (AP) score. To be noted, the AP score was merely used to compare the performance of different models, rather than estimating how self-supervised learning can approximate MSA. This is because the phastCons scores were derived from 100 species while our model was pretrained on only 72 species.

4 Estimating splice site strength from RNA-seq samples

SpliSER (v0.1.7) (Dent et al., 2021) was utilized to assess the splice site strength ("splice site strength estimation", SSE) in RNA-seq samples of the K562 cell line. The alignment files (in bam format, GRCh38/hg38) of reversely stranded polyA plus RNA-seq or total RNA-seq were downloaded from the ENCODE project(ENCODE Project Consortium, 2012; Luo et al., 2020) (accession IDs listed in Supplementary Table S4). We removed unmapped or multi-mapped reads (MAPQ $<$ 255) (Dobin & Gingeras, 2015) from the alignment files by samtools (v1.10)65 and then calculated the SSE of splice sites by regtools (v0.5.2)(Cotto et al., 2021) and SpliSER. Only the splice sites identified in at least 3 samples were reserved and the median SSE value was taken to annotate the splice sites included in the GENCODE annotation (v41, GRCh38/hg38).

5 Analysis of attention weights at alternative spliced sites

The percent splice in (PSI or Ψ) values of donor and acceptor sites in 50 tissues from the GTEx project (Consortium, 2015) were downloaded from the SpliceMap database (Wagner et al., 2023). Since the Ψ values of alternative splice sites may vary in different tissues, we retained only the sites with consistent trends in most ($>$ 66.7 %) tissues, resulting in 5978 and 4475 sites for 5' and 3' splice sites, respectively, and took the Ψ in whole blood as the representative value. The alternative splices were then divided into 2 groups based on the Ψ value in whole blood, where the sites with $\Psi > 0.5$ were labelled as "strong" and the others as "weak", and the attention weights of the sites in the two groups were compared (Figure S8). The attention weights in the "strong" group were significantly higher than those in the "weak" group (Mann-Whitney U test, $p < 1 \times 10^{-16}$ for both 5' and 3' splice sites, and Cohen's D effect(Sullivan & Feinn, 2012) of log-transformed attention weights was 0.46 and 1.07, respectively).

6 In silico mutagenesis analysis

In silico mutagenesis (ISM)(Zhou & Troyanskaya, 2015) was employed to investigate the effects of genetic variants on their neighboring nucleotides or branchpoint/splice site predictions. Specifically, we enumerated all possible single nucleotide variants (SNVs) in whole or a custom subregion of each input sequence. Only a single SNV is introduced to a sequence each time. The sequences without and with variants are referred to as wild-type (WT) and mutant (MT) sequences, respectively. The difference between the predictions given by the model for MT and WT sequences can be used to estimate the effects of variants.

7 Visualization of consensus sequences

We visualized the consensus sequences around branchpoints and splice sites using the Logomaker package(Tareen & Kinney, 2020). The logarithm of the ratio between observed and expected nucleotide frequency ($\log_2 \frac{\mathrm{OF}}{\mathrm{EF}}$) was taken for visualization. The observed frequencies (OF) at each position were

obtained by taking the average occurrence of nucleotide types across different sequences. The expected frequency (EF) of each nucleotide type (background frequency) was obtained by taking the average occurrence frequency of entire sequences.

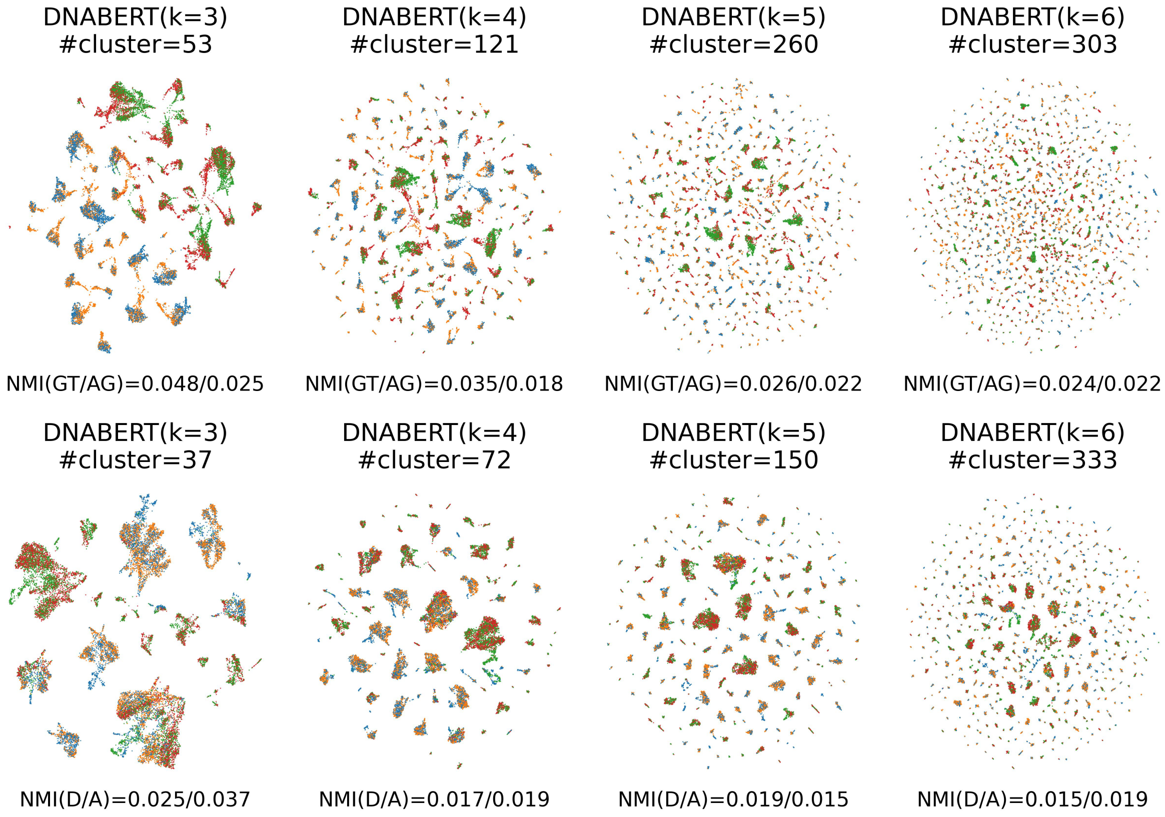

Figure S1: Comparison of UMAP visualization of embeddings of (**up**) splice/non-spilce sites and (**down**) strong/weak splice sites generated by DNABERT with different token length (k=3, 4, 5, 6). The Leiden algorithm was employed to cluster the embeddings. The normalized mutual information (NMI) score was calculated based on Leiden (Traag et al., 2019) clustering.

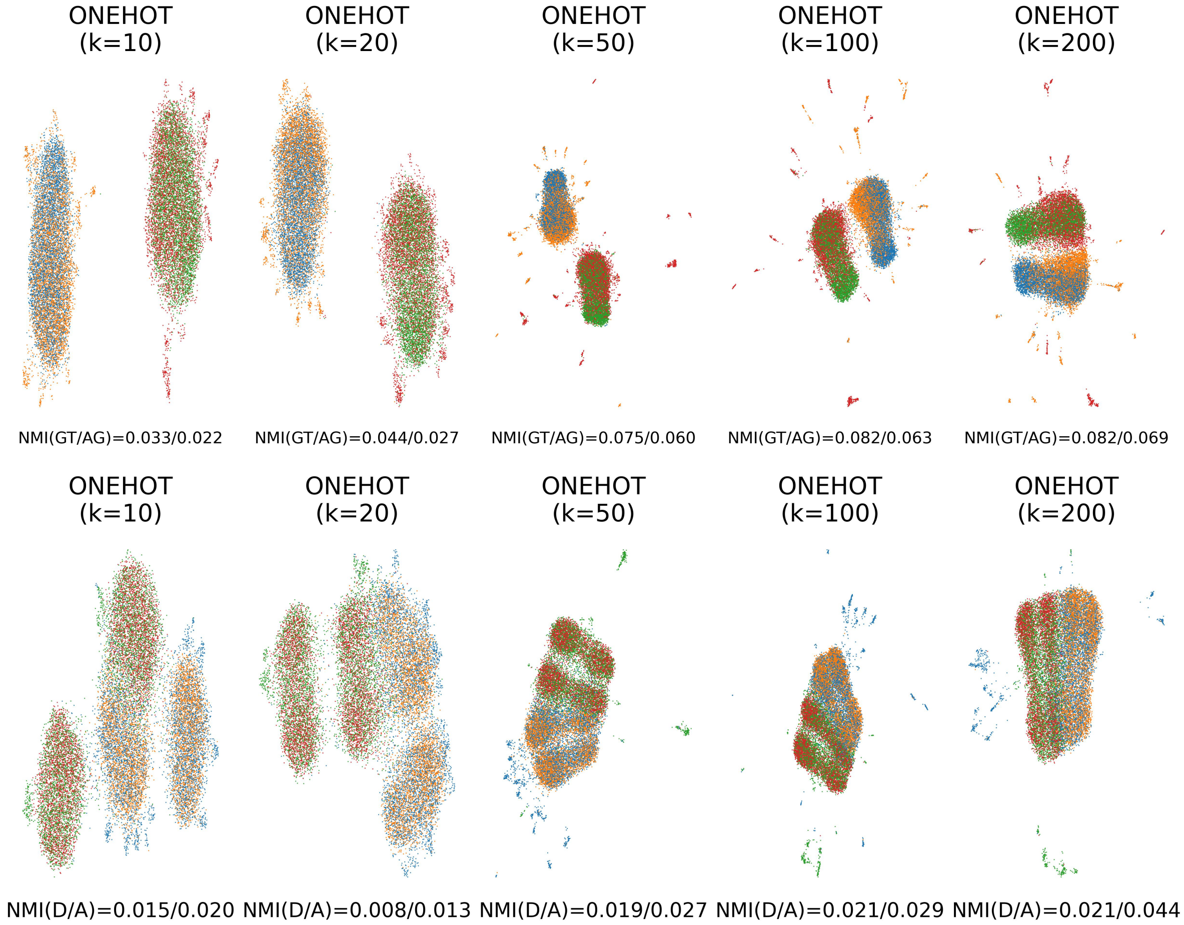

Figure S2: UMAP of (**up**) splice/non-splice site and (**down**) strong/weak splice site embeddings obtained by one-hot encoded sequences of different length. The Leiden algorithm was employed to cluster the embeddings.

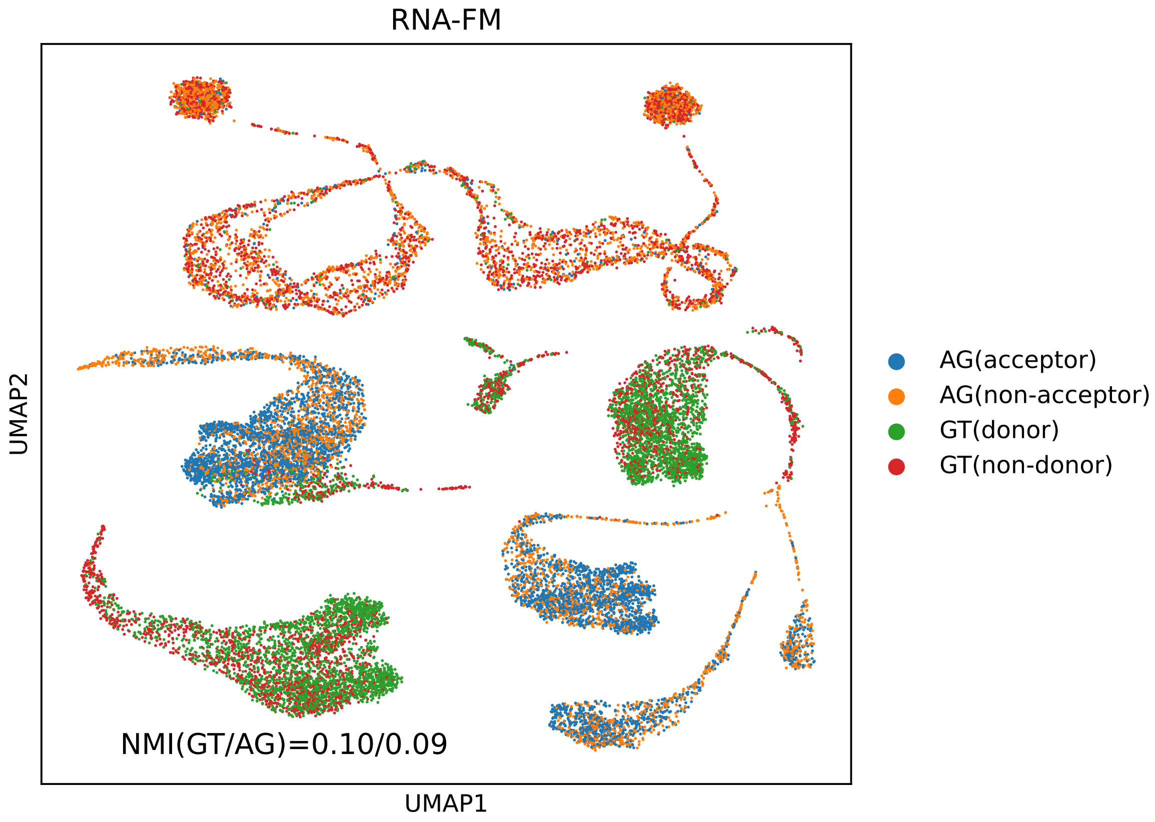

Figure S3: UMAP of splice site embeddings generated by RNA-FM

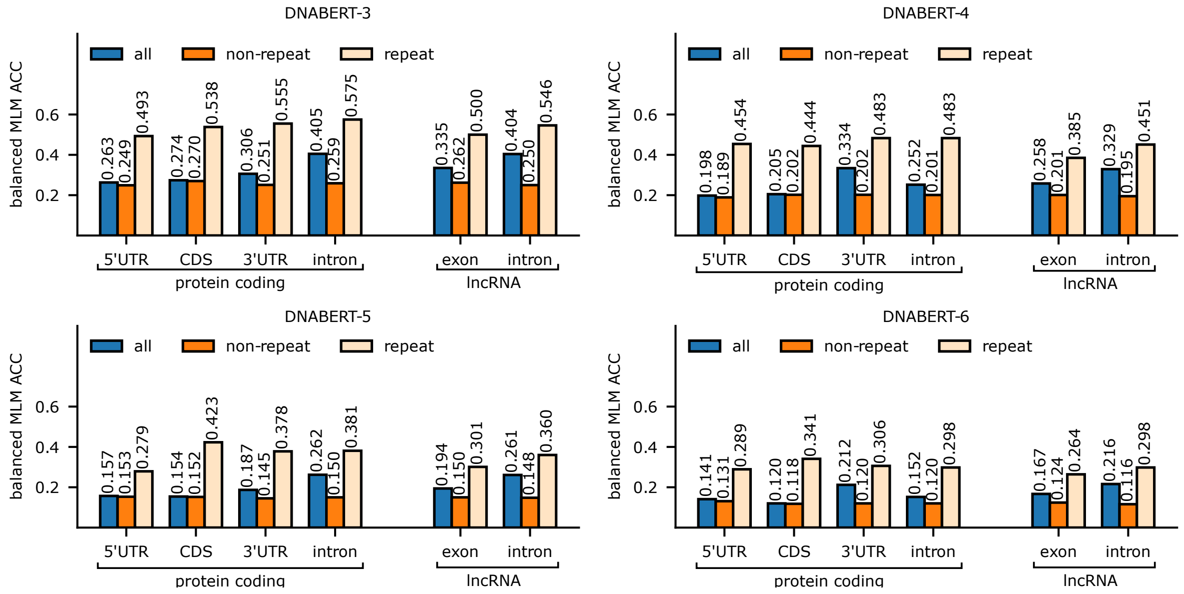

Figure S4: Balanced accuracy for masked token prediction of DNABERT in different regions. (The accuracy decreased with the increase of the length of the token. This is because the vocabulary size increases with the length of the token, making it harder for the model to predict the masked tokens.)

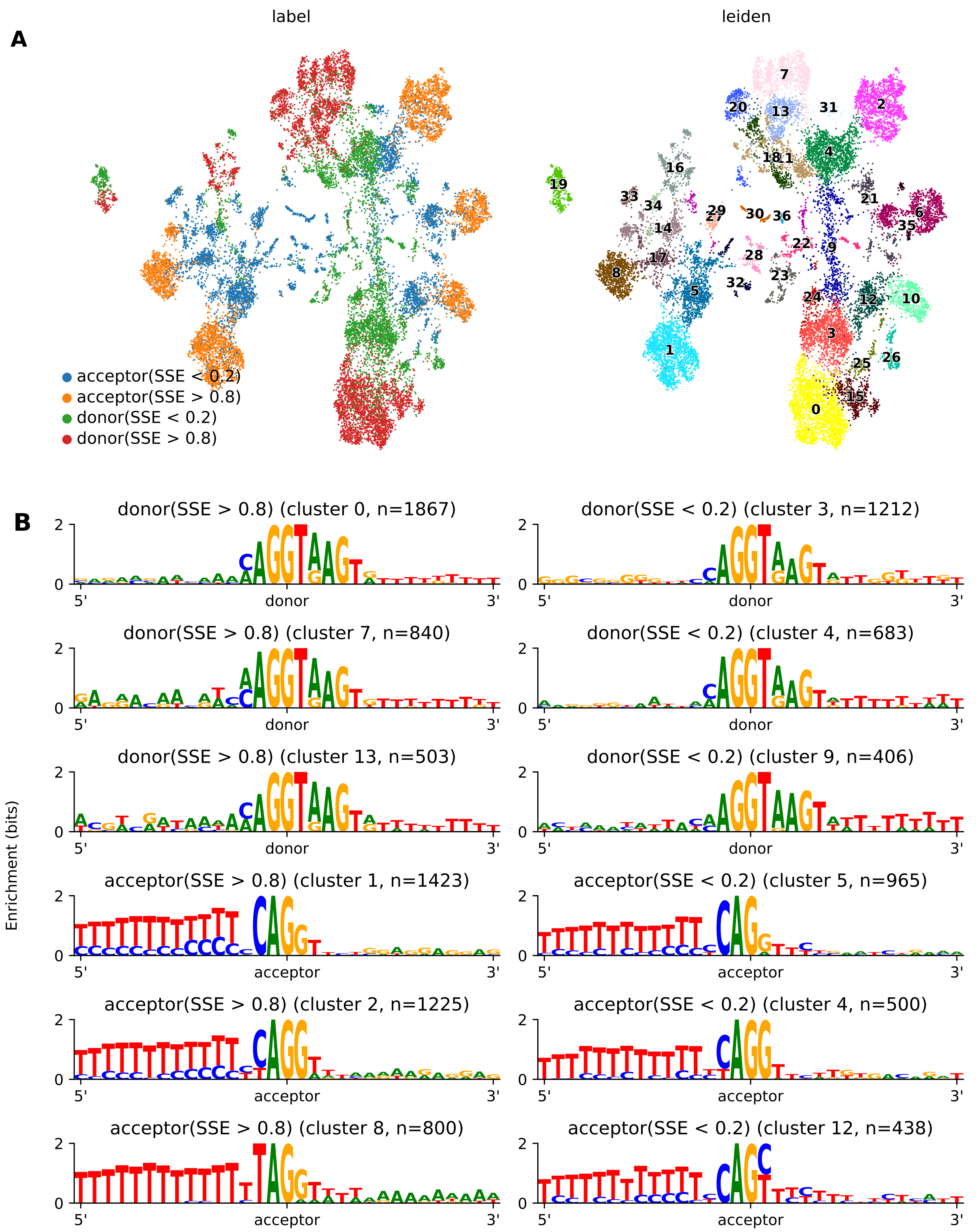

Figure S5: Analysis of splice sites in different clusters (**A**) UMAP of splice sites colored by Leiden clusters. (**B**) The sequence logo of the splice sites in different clusters (only the top-3 clusters are shown).

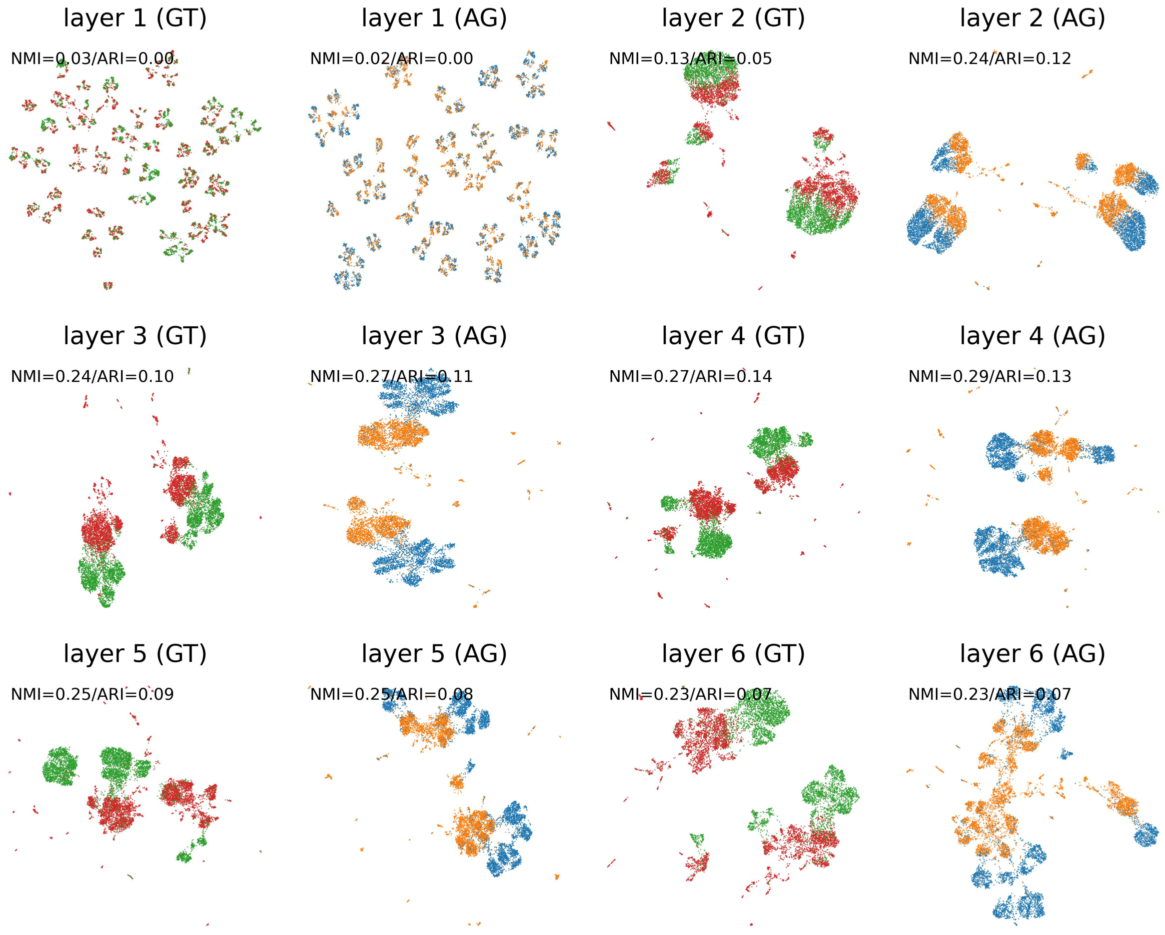

Figure S6: Comparison of nucleotide embeddings from different Transformer encoder layers in SpliceBERT

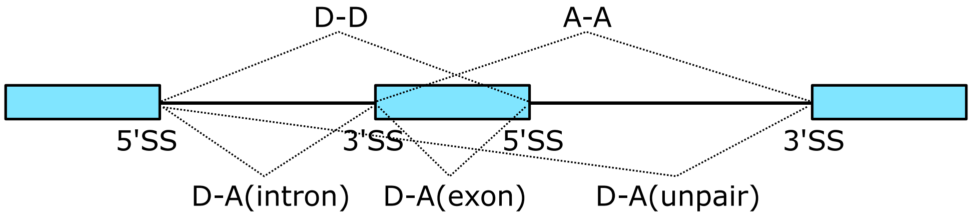

Figure S7: Different combination of donor/acceptor sites

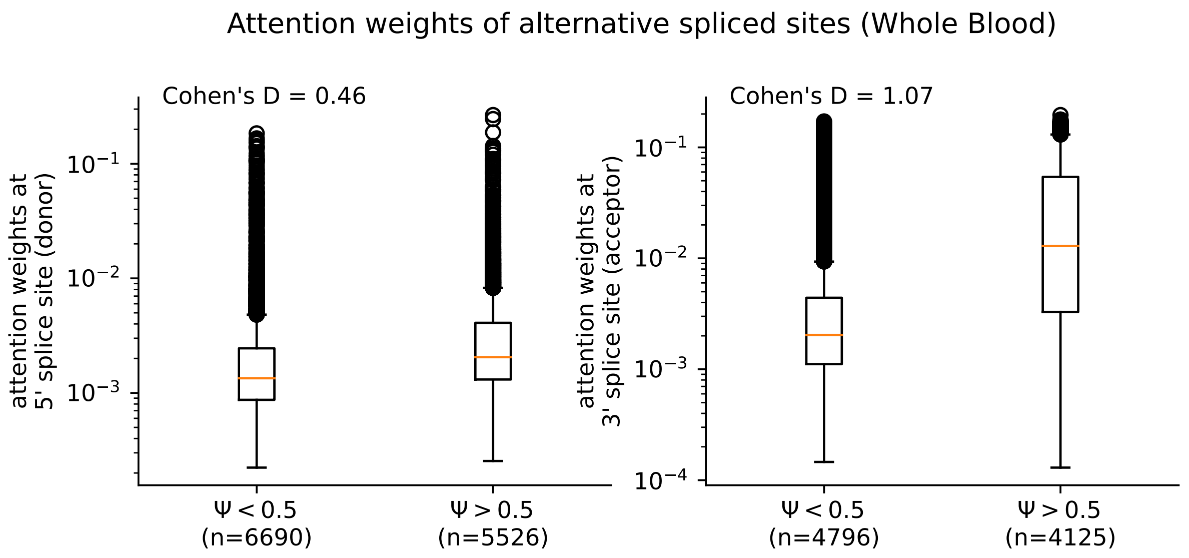

Figure S8: Analysis of attention weights of 5' and 3' alternative spliced sites in Whole Blood. Cohen's D effect between attention weights of low Ψ ($\Psi < 0.5$) and high Ψ ($\Psi > 0.5$) group for 5'splice and 3'splice site was 0.46 and 1.07, respectively and the P-value (by Mann-Whitney U test) of both group are below 10^{-16}.

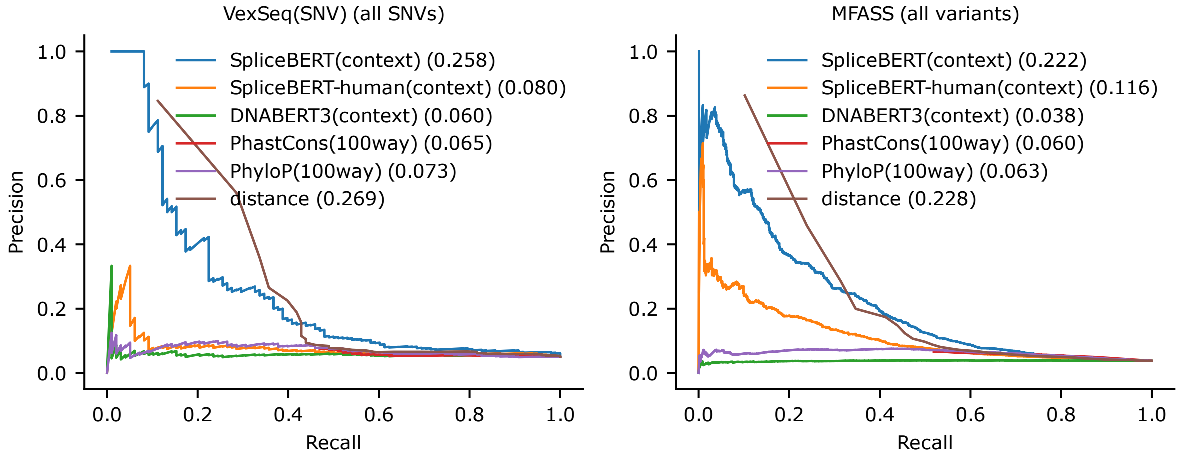

Figure S9: Precision-recall curves of SpliceBERT and baseline methods for zero-shot prediction of variant effects on RNA splicing. When all variants are considered, the distance (distance from splice sites to variants) dominates the performance because variants residing in splice sites are very likely to largely impact splicing.

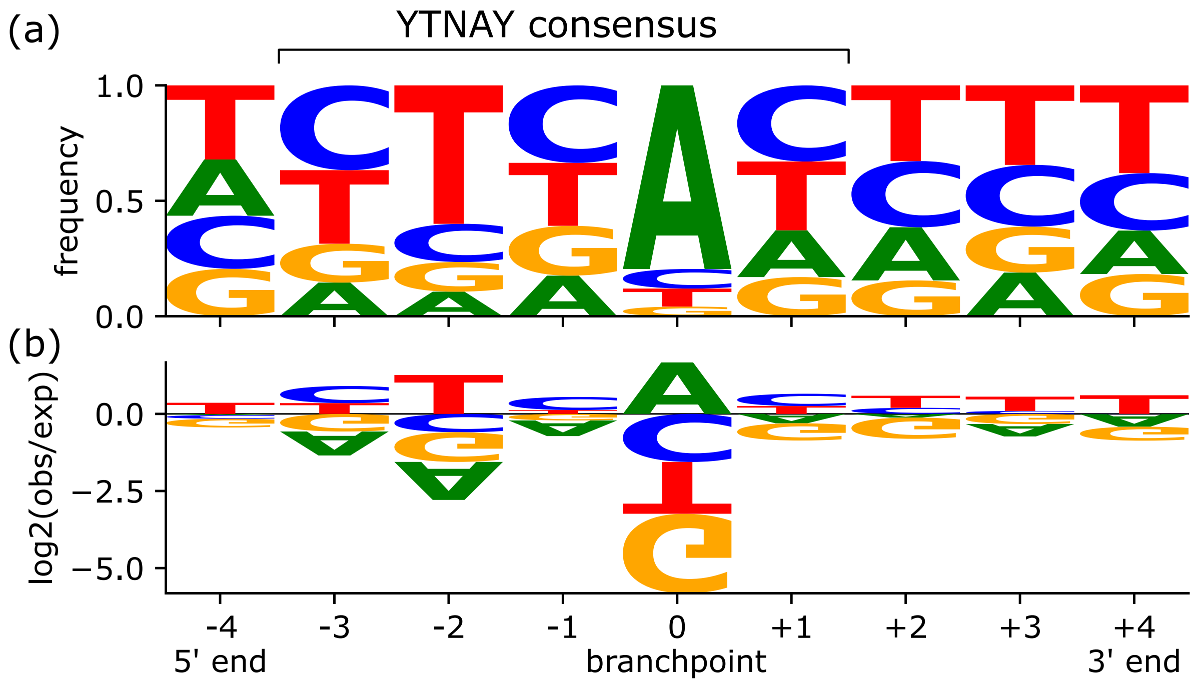

Figure S10: Visualization of the consensus sequences around branchpoints the Mercer dataset. The position-specific frequency of the nucleotide type around branchpoint sites is shown in (a) original scale or (b) log2(frequency/expected frequency) scale, respectively. There is a YTNAY consensus around the branchpoint site (Y: C/T, N: A/C/G/T)(Gao et al., 2008).

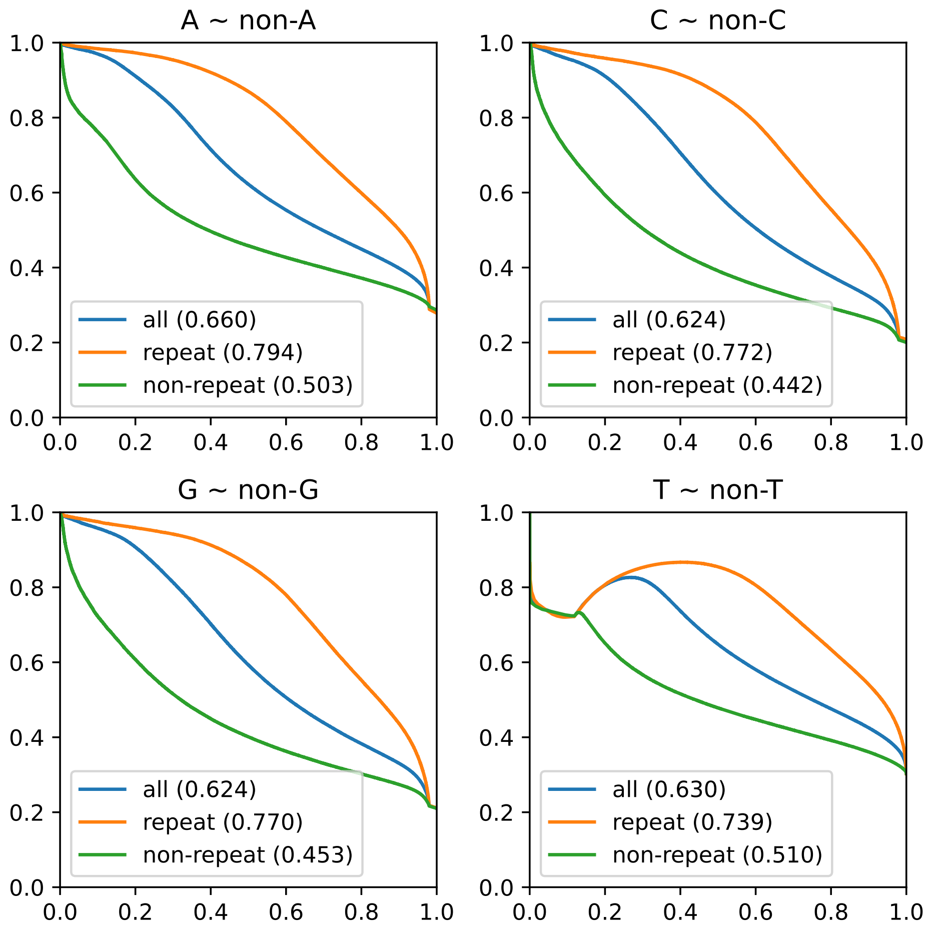

Figure S11: Precision-recall curves of SpliceBERT for masked token prediction in repeat/non-repeat regions for A, C, G, T, respectively.

References

Chen, K. M., Cofer, E. M., Zhou, J., & Troyanskaya, O. G. (2019, April). Selene: a PyTorch-based deep learning library for sequence data. *Nat Methods*, *16*(4), 315–318. Retrieved 2022-08-29, from https://www.nature.com/articles/s41592-019-0360-8 doi: 10.1038/s41592-019-0360-8

Consortium, T. G. (2015, May). The Genotype-Tissue Expression (GTEx) pilot analysis: Multitissue gene regulation in humans. *Science*, *348*(6235), 648–660. Retrieved 2019-08-12, from https://science.sciencemag.org/content/348/6235/648 doi: 10.1126/science.1262110

Cotto, K. C., Feng, Y.-Y., Ramu, A., Skidmore, Z. L., Kunisaki, J., Richters, M., ... Griffith, M. (2021, April). *RegTools: Integrated analysis of genomic and transcriptomic data for the discovery of splicing variants in cancer.* bioRxiv. Retrieved 2022-07-27, from https://www.biorxiv.org/content/10.1101/436634v5 doi: 10.1101/436634

Dent, C. I., Singh, S., Mukherjee, S., Mishra, S., Sarwade, R. D., Shamaya, N., ... Balasubramanian, S. (2021, June). Quantifying splice-site usage: a simple yet powerful approach to analyze splicing. *NAR Genomics and Bioinformatics*, *3*(2), lqab041. Retrieved 2022-07-25, from https://doi.org/10.1093/nargab/lqab041 doi: 10.1093/nargab/lqab041

Dobin, A., & Gingeras, T. R. (2015, September). Mapping RNA-seq Reads with STAR. *Curr Protoc Bioinformatics*, *51*, 11.14.1–11.14.19. Retrieved 2019-03-08, from https://www.ncbi.nlm.nih.gov/pmc/articles/PMC4631051/ doi: 10.1002/0471250953.bi1114s51

ENCODE Project Consortium. (2012, September). An integrated encyclopedia of DNA elements in the human genome. *Nature*, *489*(7414), 57–74. doi: 10.1038/nature11247

Gao, K., Masuda, A., Matsuura, T., & Ohno, K. (2008, April). Human branch point consensus sequence is yUnAy. *Nucleic Acids Research*, *36*(7), 2257–2267. Retrieved 2022-12-11, from https://doi.org/10.1093/nar/gkn073 doi: 10.1093/nar/gkn073

Haeussler, M., Zweig, A. S., Tyner, C., Speir, M. L., Rosenbloom, K. R., Raney, B. J., ... Kent, W. J. (2019). The UCSC Genome Browser database: 2019 update. *Nucleic Acids Res.*, *47*(D1), D853–D858. doi: 10.1093/nar/gky1095

Li, H. (2011, March). Tabix: fast retrieval of sequence features from generic TAB-delimited files. *Bioinformatics*, *27*(5), 718–719. Retrieved 2023-06-02, from https://doi.org/10.1093/bioinformatics/btq671 doi: 10.1093/bioinformatics/btq671

Luo, Y., Hitz, B. C., Gabdank, I., Hilton, J. A., Kagda, M. S., Lam, B., ... Cherry, J. M. (2020, January). New developments on the Encyclopedia of DNA Elements (ENCODE) data portal. *Nucleic Acids Res*, *48*(D1), D882–D889. doi: 10.1093/nar/gkz1062

Pollard, K. S., Hubisz, M. J., Rosenbloom, K. R., & Siepel, A. (2010, January). Detection of nonneutral substitution rates on mammalian phylogenies. *Genome Res*, *20*(1), 110–121. Retrieved 2018-01-27, from https://www.ncbi.nlm.nih.gov/pmc/articles/PMC2798823/ doi: 10.1101/gr.097857.109

Ramírez, F., Ryan, D. P., Grüning, B., Bhardwaj, V., Kilpert, F., Richter, A. S., ... Manke, T. (2016, July). deepTools2: a next generation web server for deep-sequencing data analysis. *Nucleic Acids Res*, *44*(Web Server issue), W160–W165. Retrieved 2020-12-29, from https://www.ncbi.nlm.nih

.gov/pmc/articles/PMC4987876/ doi: 10.1093/nar/gkw257

Siepel, A., Bejerano, G., Pedersen, J. S., Hinrichs, A. S., Hou, M., Rosenbloom, K., … Haussler, D. (2005, August). Evolutionarily conserved elements in vertebrate, insect, worm, and yeast genomes. *Genome Res*, *15*(8), 1034–1050. Retrieved 2023-03-15, from https://www.ncbi.nlm.nih.gov/pmc/articles/PMC1182216/ doi: 10.1101/gr.3715005

Su, J. (2020, Dec). *Hierarchical decomposition of positional encoding enables BERT to handle longer sequences (in Chinese).* Retrieved from https://spaces.ac.cn/archives/7947

Sullivan, G. M., & Feinn, R. (2012). Using effect size—or why the p value is not enough. *Journal of graduate medical education*, *4*(3), 279–282.

Tareen, A., & Kinney, J. B. (2020, April). Logomaker: beautiful sequence logos in Python. *Bioinformatics*, *36*(7), 2272–2274. Retrieved 2022-12-21, from https://doi.org/10.1093/bioinformatics/btz921 doi: 10.1093/bioinformatics/btz921

Traag, V. A., Waltman, L., & van Eck, N. J. (2019, March). From Louvain to Leiden: guaranteeing well-connected communities. *Sci Rep*, *9*(1), 5233. Retrieved 2022-03-28, from https://www.nature.com/articles/s41598-019-41695-z doi: 10.1038/s41598-019-41695-z

Wagner, N., Çelik, M. H., Hölzlwimmer, F. R., Mertes, C., Prokisch, H., Yépez, V. A., & Gagneur, J. (2023, May). Aberrant splicing prediction across human tissues. *Nat Genet*, 1–10. Retrieved 2023-05-06, from https://www.nature.com/articles/s41588-023-01373-3 doi: 10.1038/s41588-023-01373-3

Zhou, J., & Troyanskaya, O. G. (2015, October). Predicting effects of noncoding variants with deep learning–based sequence model. *Nat Methods*, *12*(10), 931–934. Retrieved 2021-09-07, from https://www.nature.com/articles/nmeth.3547 doi: 10.1038/nmeth.3547
