# Supplementary material for: Self-supervised learning on millions of primary RNA sequences from 72 vertebrates improves sequence-based RNA splicing prediction
Source: Brief Bioinform. 2024 Apr 11;25(3):bbae163. doi: 10.1093/bib/bbae163 (PMC11009468; doi:10.1093/bib/bbae163)
Supplement: SpliceBERT_Tables_BIB_v1_bbae163 [file splicebert_tables_bib_v1_bbae163.docx]

**Table S1. Statistics of attention weights around donors and acceptors**

Table S1a. Testing the difference in attention weights between different donor/acceptor pairs (Cohen'D effect size)

|  | **D-A(intron)** |
| --- | --- |
| **D-A(exon)** | 1.04 |
| **D-A(unpair)** | 0.855 |
| **D-D** | 0.835 |
| **A-A** | 0.885 |
| **random pair** | 2.15 |

Table S1b. The distribution of attention weights around donors and acceptors

| **group** | **layer** | **average attention in acceptor*** | **background** | **fold change** |
| --- | --- | --- | --- | --- |
| donors' attention on around acceptors | 1 | 0.0022 | 0.0032 | 0.6828 |
|  | 2 | 0.0014 | 0.0026 | 0.5433 |
|  | 3 | 0.0051 | 0.0028 | 1.8636 |
|  | 4 | 0.0443 | 0.0028 | 16.0956 |
|  | 5 | 0.0043 | 0.0025 | 1.6813 |
|  | 6 | 0.002 | 0.0028 | 0.7277 |
| acceptors' attention on around donors | **layer** | **average attention in donor*** | **background** | **fold change** |
|  | 1 | 0.0028 | 0.0034 | 0.8328 |
|  | 2 | 0.0017 | 0.0025 | 0.6631 |
|  | 3 | 0.0035 | 0.0028 | 1.2215 |
|  | 4 | 0.1067 | 0.0046 | 23.3414 |
|  | 5 | 0.0075 | 0.0027 | 2.716 |
|  | 6 | 0.0033 | 0.0032 | 1.0173 |
| *: 2nt in intron and 1nt in exon | | |  |  |

**Table S2: F1-score of SpliceBERT and baseline models for predicting splice sites across species**

Table S2a: F1 score

| **Species** | **type** | **Spliceator** | **SpliceFinder** | **DSSP** | **MaxEntScan** | **NNSplice** | **SpliceAI (400nt)** | **DNABERT** | **SpliceBERT (w/o PT)** | **SpliceBERT-human** | **SpliceBERT** |
| --- | --- | --- | --- | --- | --- | --- | --- | --- | --- | --- | --- |
| **zebrafish** | donor | 0.951 | 0.883 | 0.944 | 0.942 | 0.753 | 0.9548 | 0.9679 | 0.9521 | 0.9647 | **0.9692** |
|  | acceptor | 0.919 | 0.918 | 0.936 | 0.856 | 0.732 | 0.9382 | 0.9330 | 0.9251 | 0.9417 | **0.9443** |
|  | average | 0.935 | 0.901 | 0.940 | 0.899 | 0.743 | 0.9465 | 0.9505 | 0.9386 | 0.9532 | **0.9568** |
| **fruit fly** | donor | 0.948 | 0.871 | 0.939 | 0.946 | 0.762 | 0.9483 | 0.9508 | 0.9466 | 0.9419 | **0.9549** |
|  | acceptor | 0.910 | 0.842 | 0.914 | 0.874 | 0.761 | 0.9096 | 0.9105 | 0.9135 | 0.9189 | **0.9373** |
|  | average | 0.929 | 0.857 | 0.927 | 0.910 | 0.762 | 0.9289 | 0.9307 | 0.9300 | 0.9418 | **0.9461** |
| **worm** | donor | 0.941 | 0.808 | 0.897 | 0.930 | 0.699 | 0.9300 | 0.9299 | 0.9101 | 0.9169 | **0.9411** |
|  | acceptor | 0.891 | 0.779 | 0.822 | 0.837 | 0.571 | 0.8895 | 0.8889 | 0.8636 | 0.8999 | **0.9274** |
|  | average | 0.916 | 0.794 | 0.860 | 0.884 | 0.635 | 0.9097 | 0.9094 | 0.8869 | 0.9084 | **0.9343** |
| **arabidopsis** | donor | 0.949 | 0.813 | 0.871 | 0.925 | 0.662 | 0.9098 | 0.9286 | 0.9146 | 0.9311 | **0.9380** |
|  | acceptor | 0.908 | 0.772 | 0.833 | 0.867 | 0.634 | 0.8984 | 0.8890 | 0.8787 | 0.9141 | **0.9343** |
|  | average | 0.929 | 0.793 | 0.852 | 0.896 | 0.648 | 0.9041 | 0.9088 | 0.8967 | 0.9226 | **0.9361** |

Table S2b: Precision

| **Species** | **type** | **Spliceator** | **SpliceFinder** | **DSSP** | **MaxEntScan** | **NNSplice** | **SpliceAI (400nt)** | **DNABERT** | **SpliceBERT (w/o PT)** | **SpliceBERT-human** | **SpliceBERT** |
| --- | --- | --- | --- | --- | --- | --- | --- | --- | --- | --- | --- |
| **zebrafish** | donor | 0.916 | 0.815 | **0.975** | 0.906 | 0.643 | 0.9561 | 0.9531 | 0.9418 | 0.9420 | 0.9480 |
|  | acceptor | 0.860 | 0.850 | **0.942** | 0.754 | 0.639 | 0.9109 | 0.9160 | 0.8950 | 0.9002 | 0.9020 |
|  | average | 0.888 | 0.833 | **0.959** | 0.830 | 0.641 | 0.9335 | 0.9346 | 0.9184 | 0.9211 | 0.9250 |
| **fruit fly** | donor | 0.916 | 0.798 | **0.966** | 0.918 | 0.653 | 0.9438 | 0.9212 | 0.9401 | 0.9102 | 0.9366 |
|  | acceptor | 0.862 | 0.728 | **0.931** | 0.795 | 0.648 | 0.8772 | 0.9192 | 0.9048 | 0.8644 | 0.8969 |
|  | average | 0.889 | 0.763 | **0.949** | 0.857 | 0.651 | 0.9105 | 0.9202 | 0.9225 | 0.8873 | 0.9168 |
| **worm** | donor | 0.914 | 0.712 | **0.970** | 0.916 | 0.619 | 0.9437 | 0.9051 | 0.9448 | 0.8631 | 0.9009 |
|  | acceptor | 0.869 | 0.641 | **0.931** | 0.768 | 0.580 | 0.8819 | 0.9076 | 0.9069 | 0.8354 | 0.8755 |
|  | average | 0.892 | 0.677 | **0.951** | 0.842 | 0.600 | 0.9128 | 0.9064 | 0.9259 | 0.8493 | 0.8882 |
| **arabidopsis** | donor | 0.918 | 0.722 | **0.965** | 0.905 | 0.572 | 0.9486 | 0.9194 | 0.9483 | 0.9011 | 0.9011 |
|  | acceptor | 0.891 | 0.631 | **0.942** | 0.792 | 0.571 | 0.9145 | 0.9347 | 0.9249 | 0.8762 | 0.8902 |
|  | average | 0.905 | 0.677 | **0.954** | 0.849 | 0.572 | 0.9316 | 0.9271 | 0.9366 | 0.8887 | 0.8957 |

Table S2c: Recall

| **Species** | **type** | **Spliceator** | **SpliceFinder** | **DSSP** | **MaxEntScan** | **NNSplice** | **SpliceAI (400nt)** | **DNABERT** | **SpliceBERT (w/o PT)** | **SpliceBERT-human** | **SpliceBERT** |
| --- | --- | --- | --- | --- | --- | --- | --- | --- | --- | --- | --- |
| **zebrafish** | donor | 0.990 | 0.964 | 0.915 | 0.981 | 0.908 | 0.9545 | 0.9837 | 0.9636 | 0.9888 | **0.9916** |
|  | acceptor | 0.987 | **0.998** | 0.929 | 0.990 | 0.856 | 0.9675 | 0.9504 | 0.9580 | 0.9875 | 0.9911 |
|  | average | 0.989 | 0.981 | 0.922 | 0.986 | 0.882 | 0.9610 | 0.9671 | 0.9608 | 0.9882 | **0.9914** |
| **fruit fly** | donor | **0.983** | 0.958 | 0.913 | 0.976 | 0.914 | 0.9534 | 0.9824 | 0.9543 | 0.9765 | 0.9742 |
|  | acceptor | 0.964 | **0.998** | 0.898 | 0.970 | 0.921 | 0.9451 | 0.9018 | 0.9232 | 0.9814 | 0.9818 |
|  | average | 0.974 | 0.978 | 0.906 | 0.973 | 0.918 | 0.9493 | 0.9421 | 0.9388 | **0.9790** | 0.9780 |
| **worm** | donor | 0.969 | 0.933 | 0.834 | 0.945 | 0.803 | 0.9183 | 0.9560 | 0.8815 | 0.9782 | **0.9856** |
|  | acceptor | 0.914 | **0.993** | 0.736 | 0.920 | 0.564 | 0.8982 | 0.8710 | 0.8264 | 0.9759 | 0.9863 |
|  | average | 0.942 | 0.963 | 0.785 | 0.933 | 0.684 | 0.9083 | 0.9135 | 0.8540 | 0.9771 | **0.9860** |
| **arabidopsis** | donor | **0.981** | 0.929 | 0.793 | 0.946 | 0.784 | 0.8776 | 0.9381 | 0.8863 | 0.9639 | 0.9785 |
|  | acceptor | 0.927 | **0.993** | 0.748 | 0.959 | 0.713 | 0.8842 | 0.8475 | 0.8389 | 0.9561 | 0.9831 |
|  | average | 0.954 | 0.961 | 0.771 | 0.953 | 0.749 | 0.8809 | 0.8928 | 0.8626 | 0.9600 | **0.9808** |

Table S2d: P-value of t-test on F1 scores between SpliceBERT and baseline methods (donor + acceptor)

|  | SpliceBERT |
| --- | --- |
| SpliceBERT-human | 0.0031 |
| SpliceBERT (w/o PT) | 0.0030 |
| DNABERT | 0.0152 |
| SpliceAI-400nt | 0.0025 |
| Spliceator | 0.0247 |
| DSSP | 0.0086 |
| MaxEntScan | 0.0073 |
| SpliceFinder | 0.0002 |
| NNSplice | 0.00001 |

* Two-side, paired t-test was performed

**Table S3: Versions of reference genomes used for pre-training SpliceBERT**

| **#** | **Species** | **Build version (UCSC)** |
| --- | --- | --- |
| 1 | Panda | ailMel1 |
| 2 | American alligator | allMis1 |
| 3 | Lizard | anoCar2 |
| 4 | Brown kiwi | aptMan1 |
| 5 | Minke whale | balAcu1 |
| 6 | Bison | bisBis1 |
| 7 | Cow | bosTau9 |
| 8 | Marmoset | calJac4 |
| 9 | Elephant shark | calMil1 |
| 10 | Dog | canFam6 |
| 11 | Guinea pig | cavPor3 |
| 12 | White rhinoceros | cerSim1 |
| 13 | Green monkey | chlSab2 |
| 14 | Sloth | choHof1 |
| 15 | Chinese hamster | criGriChoV2 |
| 16 | Zebrafish | danRer11 |
| 17 | Armadillo | dasNov3 |
| 18 | Kangaroo rat | dipOrd1 |
| 19 | Tenrec | echTel2 |
| 20 | Horse | equCab3 |
| 21 | Hedgehog | eriEur2 |
| 22 | Cat | felCat9 |
| 23 | Fugu | fr3 |
| 24 | Atlantic cod | gadMor1 |
| 25 | Chicken | galGal6 |
| 26 | Malayan flying lemur | galVar1 |
| 27 | Stickleback | gasAcu1 |
| 28 | Medium ground finch | geoFor1 |
| 29 | Gorilla | gorGor6 |
| 30 | Naked mole-rat | hetGla2 |
| 31 | Homo Sapiens | hg38 |
| 32 | Coelacanth | latCha1 |
| 33 | Elephant | loxAfr3 |
| 34 | Crab-eating macaque | macFas5 |
| 35 | Turkey | melGal5 |
| 36 | Budgerigar | melUnd1 |
| 37 | Mouse lemur | micMur2 |
| 38 | Mouse | mm39 |
| 39 | Opossum | monDom5 |
| 40 | Ferret | musFur1 |
| 41 | Microbat | myoLuc2 |
| 42 | Tibetan frog | nanPar1 |
| 43 | Gibbon | nomLeu3 |
| 44 | Pika | ochPri3 |
| 45 | Nile tilapia | oreNil2 |
| 46 | Platypus | ornAna2 |
| 47 | Rabbit | oryCun2 |
| 48 | Medaka | oryLat2 |
| 49 | Bushbaby | otoGar3 |
| 50 | Sheep | oviAri4 |
| 51 | Chimpanzee | panTro6 |
| 52 | Baboon | papAnu4 |
| 53 | Lamprey | petMar2 |
| 54 | Orangutan | ponAbe3 |
| 55 | Rock hyrax | proCap1 |
| 56 | Megabat | pteVam1 |
| 57 | Rhesus | rheMac10 |
| 58 | Golden snub-nosed monkey | rhiRox1 |
| 59 | Rat | rn7 |
| 60 | Squirrel monkey | saiBol1 |
| 61 | Tasmanian devil | sarHar1 |
| 62 | Shrew | sorAra2 |
| 63 | Squirrel | speTri2 |
| 64 | Pig | susScr11 |
| 65 | Zebra finch | taeGut2 |
| 66 | Tarsier | tarSyr2 |
| 67 | Tetraodon | tetNig2 |
| 68 | Garter snake | thaSir1 |
| 69 | Manatee | triMan1 |
| 70 | Tree shrew | tupBel1 |
| 71 | Alpaca | vicPac2 |
| 72 | African clawed frog | xenLae2 |

**Table S4: K562 RNA-seq samples from the ENCODE project**

| **experiment ID** | **assay type** | **biological replicate** | **technical replicate** | **BAM accession ID** |
| --- | --- | --- | --- | --- |
| ENCSR000AEM | polyA plus RNA-seq | 1 | 1_1 | ENCFF754JEN |
| ENCSR000AEM | polyA plus RNA-seq | 2 | 2_1 | ENCFF525NYR |
| ENCSR000AEO | polyA plus RNA-seq | 1 | 1_1 | ENCFF864OUS |
| ENCSR000AEO | polyA plus RNA-seq | 2 | 2_1 | ENCFF696CSX |
| ENCSR000CPH | polyA plus RNA-seq | 1 | 1_1 | ENCFF950KXS |
| ENCSR000CPH | polyA plus RNA-seq | 2 | 2_1 | ENCFF828HER |
| ENCSR545DKY | polyA plus RNA-seq | 1 | 1_1 | ENCFF894FXL |
| ENCSR545DKY | polyA plus RNA-seq | 2 | 2_1 | ENCFF480JFI |
| ENCSR000AEL | total RNA-seq | 1 | 1_1 | ENCFF595XJM |
| ENCSR000AEL | total RNA-seq | 2 | 2_1 | ENCFF724WTD |
| ENCSR000AEN | total RNA-seq | 1 | 1_1 | ENCFF219JME |
| ENCSR000AEN | total RNA-seq | 2 | 2_1 | ENCFF704RNX |
| ENCSR109IQO | total RNA-seq | 1 | 1_1 | ENCFF349JGI |
| ENCSR109IQO | total RNA-seq | 2 | 2_1 | ENCFF517WTR |
| ENCSR792OIJ | total RNA-seq | 1 | 1_1 | ENCFF932MJL |
| ENCSR792OIJ | total RNA-seq | 2 | 2_1 | ENCFF170FKF |
| ENCSR885DVH | total RNA-seq | 1 | 1_1 | ENCFF992CSK |
| ENCSR885DVH | total RNA-seq | 2 | 2_1 | ENCFF548MVZ |

**Table S5: Comparison with RNA-FM (mature non-coding RNA langauge model)**

Splice site prediction, F1 score

| **Species** | **type** | **RNA-FM** | **SpliceBERT** |
| --- | --- | --- | --- |
| **zebrafish** | donor | 0.9538 | **0.9692** |
|  | acceptor | 0.9202 | **0.9443** |
|  | average | 0.9370 | **0.9568** |
|  |  |  |  |
| **fruit fly** | donor | 0.9385 | **0.9549** |
|  | acceptor | 0.8970 | **0.9373** |
|  | average | 0.9178 | **0.9461** |
|  |  |  |  |
| **worm** | donor | 0.9166 | **0.9411** |
|  | acceptor | 0.8735 | **0.9274** |
|  | average | 0.8950 | **0.9343** |
|  |  |  |  |
| **arabidopsis** | donor | 0.9075 | **0.9380** |
|  | acceptor | 0.8694 | **0.9343** |
|  | average | 0.8884 | **0.9361** |
|  |  |  |  |
| branchpoint prediction |  |  |  |
|  | **metrics** | **RNA-FM** | **SpliceBERT** |
| **human branchpoint** | AUC | 0.9490 | **0.9610** |
|  | AP | 0.6850 | **0.7450** |

**Table S6: Summary of datasets and experiment settings**

| **Task** | **Supervised?** | **Data/dataset** | **Data partition** | **Metrics** | **Baselines** | **Notes** |
| --- | --- | --- | --- | --- | --- | --- |
| Masked language modeling (pre-training) | No (self-supervised learning) | Vertebrate (n=72) primary RNA sequences (unspliced transcripts) | Random train/validation split | Not applicable | Not applicable | Masked language modeling on 72 vertebrates |
| MLM^a^ accuracy evaluation | Not applicable | Tokens (nucleotides) been masked in primary RNA sequences | Not applicable | Balanced accuracy (BACC) | Not applicable (comparing MLM accuracy in repeat and non-repeat regions.) | Evaluating the accuracy of masked language modeling in different genomic regions. |
| Conserved/non-conserved sites classification | Yes | Randomly sampled sites on human transcripts, phastCons100way conservation scores | Random train/test split | Average precision | SpliceBERT-human,  one-hot encoding | Fitting a logistic regression (LR) model based one nucleotide embeddings to classify conserved/non-conserved sites. A validation dataset was not used here because we always fitted the LR model with default parameters and no hyperparameter-tuning step was performed. |
| Splice site embedding analysis | Not applicable | Positive samples: Canonical splice sites (GT/AG) annotate in GENCODE.v41lift37 gene annotation  Negative samples: non-splice sites with GT/AG motif and MaxEntScan score above 3 | Not applicable | Normalized mutual information (NMI) | SpliceBERT-human,  DNABERT,  one-hot encoding |  |
| Attention weights analysis | Not applicable | Donor/acceptor/random site pairs within 800nt of each other in primary RNA sequences | Not applicable | Cohen’s D effect size, P-value (t-test) | Not applicable (comparing attention weights between splice donor/acceptor and random sites) |  |
| Variant effect prediction (zero-shot) | No (zero-shot prediction) | Variants from MFASS (PMID: 30503770) and vex-seq (PMID: 29859120) | Not applicable | Average precision | SpliceBERT-human,  DNABERT,  phastCons100way,  phyloP100way,  distance | The predicted scores for variant effect were converted to their quantiles for normalization. |
| Splice site prediction | Yes | Datasets taken from Spliceator’s paper  (PMID: 34814826) | 10-fold CV^b^ | F1 score | SpliceBERT-human,  DNABERT,  SpliceBERT (w/o PT^c^),  SpliceAI-400nt  Spliceator, SpliceFinder,  DSSP, MaxEntScan, NNSplice | The F1 scores of Spliceator, SpliceFinder, DSSP, MaxEntScan and NNSplice were directly taken from Spliceator’s paper because we used identical dataset provided in the paper. For a fair comparison, DNABERT and SpliceAI-400nt were retrained on the same datasets as our model. We followed the settings described in their papers to fine-tuning/train the models. Since DNABERT has multiple models with different k-mer tokenization, we found the best performance was achieved by DNABERT-3. |
| Branchpoint prediction | Yes | Datasets taken from Mercer’s paper  (PMID: 25561518) | 10-fold CV | Average precision | SpliceBERT-human,  DNABERT,  SpliceBERT (w/o PT),  LaBranchoR,  Branchpointer | For a fair comparison, LaBranchoR was re-trained on the same dataset as SpliceBERT. We did not re-train Branchpointer because it is a ready-to-use package provided in Bioconductor (<https://bioconductor.org/packages/release/bioc/html/branchpointer.html>). |

^a^ MLM: masked language modeling

^b^ CV: cross-validation

^c^ PT: pre-training
